# The B7-1 Cytoplasmic Tail Enhances Intracellular Transport and Mammalian Cell Surface Display of Chimeric Proteins in the Absence of a Linear ER Export Motif

**DOI:** 10.1371/journal.pone.0075084

**Published:** 2013-09-20

**Authors:** Yi-Chieh Lin, Bing-Mae Chen, Wei-Cheng Lu, Chien-I Su, Zeljko M. Prijovich, Wen-Chuan Chung, Pei-Yu Wu, Kai-Chuan Chen, I-Chiao Lee, Ting-Yi Juan, Steve R. Roffler

**Affiliations:** Institute of Biomedical Sciences, Academia Sinica, Taipei, Taiwan; Tohoku University, Japan

## Abstract

Membrane-tethered proteins (mammalian surface display) are increasingly being used for novel therapeutic and biotechnology applications. Maximizing surface expression of chimeric proteins on mammalian cells is important for these applications. We show that the cytoplasmic domain from the B7-1 antigen, a commonly used element for mammalian surface display, can enhance the intracellular transport and surface display of chimeric proteins in a Sar1 and Rab1 dependent fashion. However, mutational, alanine scanning and deletion analysis demonstrate the absence of linear ER export motifs in the B7 cytoplasmic domain. Rather, efficient intracellular transport correlated with the presence of predicted secondary structure in the cytoplasmic tail. Examination of the cytoplasmic domains of 984 human and 782 mouse type I transmembrane proteins revealed that many previously identified ER export motifs are rarely found in the cytoplasmic tail of type I transmembrane proteins. Our results suggest that efficient intracellular transport of B7 chimeric proteins is associated with the structure rather than to the presence of a linear ER export motif in the cytoplasmic tail, and indicate that short (less than ~ 10-20 amino acids) and unstructured cytoplasmic tails should be avoided to express high levels of chimeric proteins on mammalian cells.

## Introduction

Membrane-tethered proteins and peptides are increasingly utilized for basic research, biotechnology and medical applications [1]. Antibodies, cytokines, major histocompatibility complex molecules, fluorescent proteins, peptides, toxins, antigens, and enzymes have been directed to and anchored on the plasma membrane of cells to reveal novel functions and properties including reduced systemic toxicity, altered in vivo distribution of drugs, creation of novel signaling receptors and inhibitors, improved in vivo cellular imaging, development of screening systems for the directed evolution of glycoproteins and human monoclonal antibodies, reshaped protein and viral immunogenicity and creation of high-resolution genetic markers [2-16]. Effective utilization of membrane-tethered proteins benefits from efficient expression of chimeric proteins on the cell surface, which in turn can be limited by slow intracellular transport [17]. Poorly transported proteins may also accumulate inside cells resulting in numerous pathological conditions [18-21].

Most membrane proteins pass through the endoplasmic reticulum (ER) and Golgi apparatus before reaching the plasma membrane. Export from the ER is a selective process that is mediated by coatomer complex II (COPII) transport vesicles that bud from sites of ER exit [22]. The COPII coat is made up of Sar I, a small GTPase, Sec23-Sec24p complex and Sec13-Sec31p complexes [23,24]. Interactions between components of the COPII transport vesicles, in particular the Sec24p subunit, and short linear amino acid sequences in the cytoplasmic domain of membrane-anchored proteins, termed ER export motifs, concentrates cargo proteins at ER exit sites and enhances cargo recruitment into COPII vesicles [25]. Several ER export motifs have been identified including di-acid, hydrophobic and aromatic motifs [26-34].

The transmembrane domain and cytoplasmic tail of the B7-1 antigen is often used to tether chimeric proteins to mammalian cells due to its ability to direct high levels of chimeric proteins to the surface of cells [17,35-46]. The B7-1 cytoplasmic domain is important for cytoskeleton-dependent redistribution and costimulatory activity of B7-1 on the plasma membrane [47,48], but little is known about the role of the B7-1 cytoplasmic domain on facilitating intracellular transport. Here we investigated the role of the B7 cytoplasmic domain in accelerated intracellular transport and surface display of chimeric proteins on mammalian cells. We show that the B7-1 cytoplasmic domain enhances the intracellular transport of chimeric proteins, but extensive deletion and mutagenesis studies did not identify the presence of linear ER export motifs in the B7-1 cytoplasmic tail. Instead, rapid intracellular transport correlated with the predicted secondary structure of cytoplasmic domains. Analysis of over one thousand human and mouse protein sequences found that many reported ER export motifs are rarely found in type I transmembrane proteins. Our results suggest that facilitated ER export of B7-1 chimeric proteins is associated with structure rather than to the presence of a linear ER export motif. Our findings may help guide the design of improved fusion proteins for expression on mammalian cells and might help explain the mechanism of certain diseases associated with intracellular protein accumulation.

## Materials and Methods

### Antibodies

Mouse monoclonal antibodies 3.3 and 36.2 against human AFP have been described [35]. Rat anti-HA (clone 3F10) was from Roche (Mannheim, Germany). Mouse anti-HA (clone 16B12) was from Covance (Berkeley, CA). Rabbit anti-BiP was from Affinity BioReagents (Golden, CO). Secondary antibodies were from Jackson Immunoresearch (West Grove, PA) and ICN Pharmaceuticals (Aurora, OH).

### Plasmids

The plasmids p2C11-B7-38, pAFP-B7-38, p2C11-PDGFR and pAFP-PDGFR have been described [35]. The B7 cytoplasmic domain was progressively deleted by PCR amplification of p2C11-B7-38 with the indicated primers (Table S1). The 2C11 scFv was replaced with the AFP gene to generate pAFP-B7-28, pAFP-B7-19, pAFP-B7-10, pAFP-B7-5 and pAFP-B7-1 which retained 28, 19, 10, 5 and 1 amino acids of the B7 cytoplasmic tail, respectively (Table S2). *A* *series* of alanine mutants (pAFP-B7-M1 to pAFP-B7-M9 in Table S1) was generated by site-directed mutagenesis using the QuikChange® Site-Directed Mutagenesis Kit (Stratagene, La Jolla, CA). Constructs with altered full-length cytoplasmic tails (pAFP-B7-S1, pAFP-B7-S2, pAFP-B7-NE, pAFP-B7-NC and pAFP-B7-CS) were generated by first performing overlap PCR with five primers (Table S1), and then performing overlap PCR with pAFP-B7-5. Other constructs listed in Table S2 were created by PCR using the primers listed in Table S1. The genes coding chimeric proteins were inserted into pLNCX (Clontech) to create the corresponding retroviral expression cassettes. Sar1WT and dominant negative Sar1[T39N] and Sar1[H79G] plasmids and Rab1WT and dominant negative Rab1 (Rab1DN) plasmids were kind gifts of Dr. David Fedida (University of British Columbia, Vancouver, Canada) and Dr. Jennifer Lippincott-Schwartz (National Institute of Child Health and Human Development, Bethesda, MD) [49,50]. The genes were fused to a HA epitope tag (Sar1WT, Sar1[T39N], Sar1[H79G]), enhanced green fluorescence protein (Sar1WT, Sar1[H79G] and Rab1WT) or mCherry (Sar1[T39N] and Rab1DN).

### Transfection

3T3 and HEK293 cells cultured overnight in DMEM medium supplemented with 10% bovine calf serum, 2.98 mg/ml HEPES, 1 mg/ml sodium bicarbonate, 100 units/ml penicillin and 100 µg/ml streptomycin in six-well plates were transfected with 5 µg plasmid DNA and 15 µl Lipofectamine^TM^ 2000 according to the manufacturer’s instructions (Invitrogen). For stable expression, 3T3 or HEK293 cells were infected with pseudotyped retroviral particles and selected in medium containing G418 as described [4]. For Sar1 and Rab1 studies, 3T3 cells that stably expressed 2C11-B7-38 [35] were transiently transfected with 2.5 µg plasmid DNA coding Sar1 or Rab1 genes using 5 µl Lipofectamine^TM^ 2000 at 48 h before the cells were analyzed by fluorescence-activated cell sorting of mCherry or eGFP intracellular fluorescence and 2C11 scFv levels on the surface of live cells.

### Immunoblotting

Western blot analysis of transfected 3T3 cells was performed as described [51]. Immunoblots were sequentially incubated with rat anti-HA and HRP-conjugated anti-rat antibody. Bands were visualized by ECL detection (Pierce, Rockford, IL).

### Flow cytometer analysis

Cells were stained with rat anti-HA antibody at 4°C, washed and then incubated with FITC-conjugated goat anti-rat antibody. Cells were suspended in PBS containing 5 µg/ml propidium iodide (Sigma) immediately before the surface immunofluorescence of 10^4^ viable cells was measured with a FACScalibur flow cytometer (Becton Dickinson, Mountain View, CA). Dead cells, identified by propidium iodide fluorescence, were gated out. Immunofluorescence staining with anti-HA antibodies was performed on live, non-permeabilized cells so only those proteins expressed at the cell surface were accessible to anti-HA antibodies and fluorescence values reflect the amount of protein expressed on the cell surface. For studies using eGFP and mCherry-labeled Sar1 or Rab1 genes, transfected cells were stained with rat anti-HA antibody at 4°C, washed and then incubated with Cy5-conjugated goat anti-rat antibody. Viable cells were identified by staining with Fixable Viability Dye eFluor® 780 (eBioscience, San Diego, CA) immediately before the surface immunofluorescence of the cells was measured on a BD LSR II flow cytometer (Becton Dickinson). Mean Cy5 immunofluorescence intensities, representing surface scFv levels, were calculated using FlowJo (Tree Star, San Carlos, CA) for cells displaying high or low eGFP or mCherry fluorescence intensities, corresponding to cells expressing high or low levels of the Sar1 and Rab1 genes, respectively.

### Protein transport rate

3T3 cell transfectants were washed 3 times with warm methionine- and cysteine-free DMEM and then pulsed at 37°C for 30 min with 150 µCi ^35^S-

[Met+Cys] pro-mix (Amersham) in DMEM supplemented with 10% dialyzed bovine calf serum. After washing with warm medium, the cells were chased at 37°C for 0, 0.5, 1, 2, 4 and 6 h with medium containing 0.94 mg/ml methionine. The cells were washed 3 times with ice-cold PBS and surface proteins were biotinylated with EZ-link^TM^ Sulfo-NHS-SS-Biotin (Pierce) on ice for 30 min. After addition of 0.19 M glycine and 25 mM Tris-HCl, pH 8.0 to quench the biotinylation reaction, the cells were washed, harvested, and lysed with solubilization buffer (20 mM sodium phosphate, pH 7.4, 0.68 M sucrose, 0.15 M NaCl, 5 mM EDTA, 1 mg/ml BSA, 1% Triton X-100, 0.1 mM phenylmethylsulfonyl fluoride and 1X protease inhibitor cocktail) at 4°C for 2 h. Cleared lysates were immunoprecipitated with anti-AFP mAb 3.3 on Protein A Sepharose CL-4B beads (Pharmacia Biotech, Uppsala, Sweden). The beads were washed once with buffer A (100 mM Tris-HCl, pH 8.0, 1% Triton X-100, 0.2% sodium-deoxycholate, 10 mM EDTA, 10 mM EGTA, 1 mg/ml BSA, 0.5 M NaCl, and protease inhibitor cocktail) and twice with buffer B (Buffer A with 0.05% SDS and 125 mM NaCl). Bound proteins were eluted in non-reducing SDS sample buffer, diluted in solubilization buffer and mixed with ImmunoPure Immobilized streptavidin agarose beads (Pierce) to collect biotin-labeled AFP proteins. Streptavidin beads were washed once each with buffer A and buffer B. Bound biotinylated proteins were eluted with reducing SDS-PAGE sample buffer and both biotinylated (surface) and non-biotinylated (intracellular) AFP proteins were run on SDS-PAGE and visualized by autoradiography. Relative band intensities were measured with a computing densitometer and analyzing with MetaMorph ^®^V6.0 software (Universal Imaging Corporation).

### Endoglycosidase H and PNGaseF digestion

Stably transfected 3T3 cells were labeled with ^35^S-

[Met+Cys] pro-mix and then chased for 0 or 1 h. The cells were solubilized, immunoprecipitated with anti-AFP mAb 3.3 and eluted with reducing SDS sample buffer. Samples were untreated or digested with 0.5 mU EndoH_f_ (NEB) or 0.5 mU PNGaseF (NEB) for 1 h at 37°C. Proteins were run on a SDS-PAGE and visualized by autoradiography.

### Carbonate extraction

Stably transfected cells (10^7^) were suspended in 1 ml 0.1 M Na _2_CO_3_, pH 11.5 on ice for 30 min. The cells were then disrupted in a Dounce homogenizer and cleared lysates were centrifuged at 100,000xg in a TLA 100.2 rotor at 4°C for 1 hour. The pellet and supernatant fractions were run on SDS-PAGE and immunoblotted with anti-AFP antibodies.

### BiP coimmunoprecipitation

1x10^7^ transiently transfected 3T3 cells were solubilized and immunoprecipitated with mouse anti-AFP antibody as described above. One tenth of the IP mixture, corresponding to 1x10^6^ cells, was separated by SDS PAGE and immunoblotted with biotin-labeled anti-HA antibody and extravidin-HRP. The remaining IP mixture was separated by SDS PAGE and then immunoblotted with rabbit anti-BiP antibody and HRP-conjugated goat anti-rabbit antibody.

### Glycosylation mapping

A plasmid coding GFP-0-B7-38 (coding a signal peptide, HA epitope tag, enhanced GFP, the B7 transmembrane and 38 amino acid cytoplasmic domain) was digested with SalI and Ale I restriction enzymes and oligonucleotides coding for an N-linked glycosylation site followed by defined numbers of amino acids were inserted (Table S3). The number of amino acids between the glycosylation site and the transmembrane domain is indicated (i.e., GFP-8-B7-38 contains 8 amino acids between the transmembrane helix and the glycosylation site). Corresponding chimeric proteins were also constructed with a truncated cytoplasmic domain (GFP-8-B7-5 to GFP-16-B7-5) and a non-structured cytoplasmic domain (GFP-8-B7-S2 to GFP-16-B7-S2).

3T3 cells were transiently transfected with plasmids coding the glycosylation chimeric proteins. Cells were collected after 48 h, run on SDS-PAGE and immunoblotted with rat anti-HA antibody and HRP conjugated anti-rat antibody followed by ECL detection. The band intensities were analyzed with MetaMorph ^®^V6.0 software (Universal Imaging Corporation). Glycosylation was confirmed by PNGaseF digestion.

### Analysis of cytoplasmic tail sequences and ER export motifs

The amino acid sequences of 984 human and 782 mouse non-redundant type I transmembrane proteins were collected from the Pubmed protein database. Proteins that had extracellular and cytoplasmic domains of at least 15 amino acids (911 human and 733 mouse sequences) were analyzed for amino acid frequency. Comparison was made between the frequency of each amino acid in the cytoplasmic and extracellular domains of the same proteins to identify amino acids over represented in cytoplasmic domains. The preference of a particular amino acid for the cytoplasmic domain as compared to the extracellular domain was calculated as:

$$\text{Preference for cytoplasmic domain (\%) = 100 * }\frac{\text{Extracellular \% - Cytoplasmic \%}}{\text{Extracellular \%}}$$

To calculate the frequency of ER export motifs, the expected random number of occurrences of a motif was calculated as described [52] based on the actual frequency of each amino acid in the sum of all amino acids present in the cytoplasmic domains of 984 human or 782 mouse non-redundant type I transmembrane proteins.

### Secondary structure analysis

Secondary structures of cytoplasmic domain polypeptides were predicted using SSPro 2.01 [53], PSIPred [54], SOPMA [55] or YASPIN [56] secondary structure prediction programs. A metric for the degree of random coil-like structure in cytoplasmic tails was estimated by dividing the predicted percentage of random coil structure by the sum of the predicted percentage of helical and extended strand structures in the cytoplasmic tails as estimated by SOPMA or the average probability (confidence scores) of these values for SSPro, PSIPred and YASPIN. A metric for the relative transport rates of the proteins was estimated by calculating the time required for 50% of the chimeric protein to attain the Golgi glycosylated form (complex carbohydrate).

### Statistical analysis

Statistical significance of differences between mean values was estimated with GraphPad Prism® Version 5 using the unpaired (independent) t-test for unequal variances. Comparison of the significance of differences in the amino acid frequencies in the extracellular and cytoplasmic domains of type I transmembrane proteins was performed using the matched pairs test. P-values of ≤ 0.05 were considered statistically significant.

## Results and Discussion

### The B7 cytoplasmic domain can enhance surface expression of a single-chain antibody

**Figure 1:**
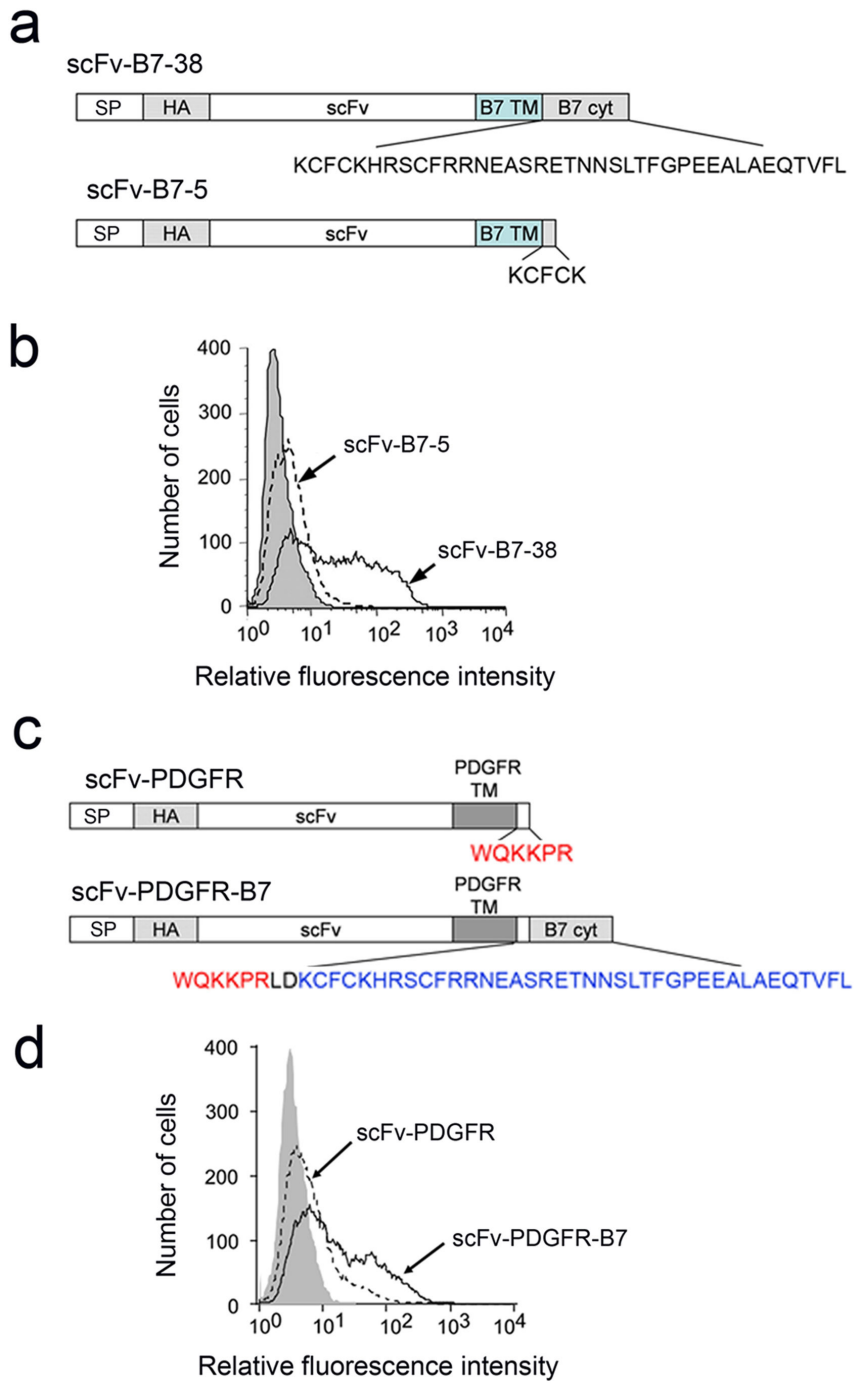
The B7 cytoplasmic domain can enhance the surface expression of a single-chain antibody. **a**) scFv-B7-38 is composed of a signal peptide (SP), a HA epitope tag, a single-chain antibody (scFv) and the B7 transmembrane domain (TM) and 38 amino acid cytoplasmic domain (cyt). The terminal 33 amino acids are deleted in scFv-B7-5. **b**) 3T3 fibroblasts transiently transfected with plasmids expressing scFv-B7-38 or scFv-B7-5 were immunofluorescence stained for the HA tag present in the scFv and analyzed on a flow cytometer. **c**) scFv-PDGFR is identical to scFv-B7-38 except that the transmembrane and truncated cytoplasmic domain is derived from the platelet derived growth factor. The B7 cytoplasmic domain was appended to the C-terminus in scFv-PDGFR-B7. **d**) 3T3 fibroblasts transiently transfected with plasmids encoding scFv-PDGFR or scFv-PDGFR-B7 were immunofluorescence stained for the HA epitope in chimeric proteins on the surface of cells and analyzed on a flow cytometer.

We previously found that single-chain antibodies (scFv) could be effectively tethered on the surface of mammalian cells by fusion to the transmembrane and cytoplasmic domains of the B7-1 protein [36]. Deletion of all but five amino acids of the B7 cytoplasmic domain in scFv-B7-5 (Figure 1a) resulted in dramatically reduced expression on the surface of transiently transfected 3T3 cells as compared to scFv-B7-38 with an intact B7 cytoplasmic domain (Figure 1b).

A single-chain antibody fused to the platelet-derived growth factor (PDGFR) transmembrane domain and first 6 amino acids of the cytoplasmic domain is poorly expressed on cells [35]. This is the membrane targeting domain present in the commercially-available pDisplay™ vector (Invitrogen). By contrast, appendage of the B7 cytoplasmic domain to the C-terminus of scFv-PDGFR (Figure 1c) enhanced surface expression (Figure 1d). We conclude that the B7 cytoplasmic domain can enhance surface expression of a chimeric protein, irrespective of the transmembrane domain.

### The B7 cytoplasmic domain enhances intracellular transport of a reporter protein

**Figure 2:**
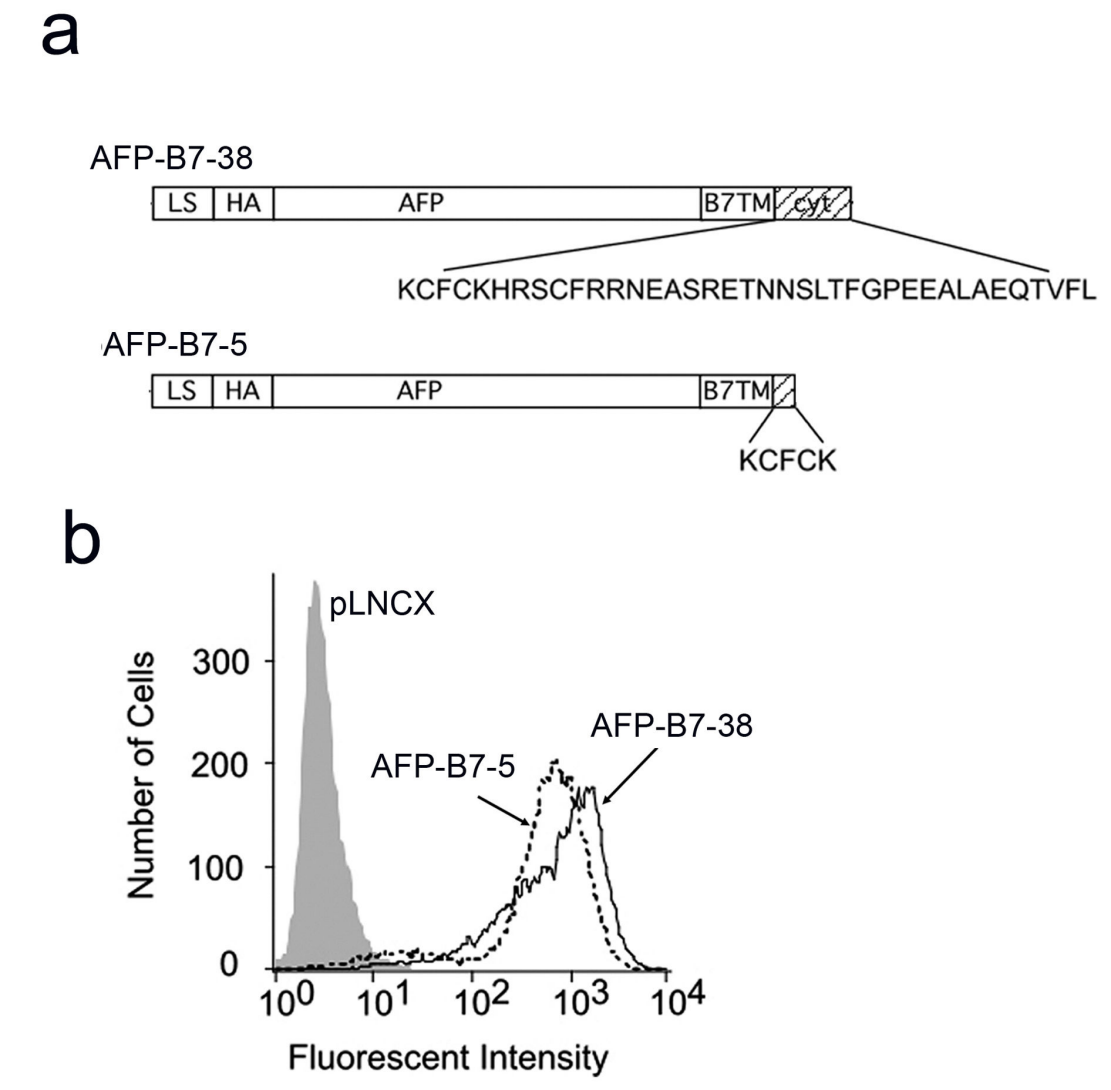
Surface display of AFP chimeric proteins is insensitive to truncation of the B7 cytoplasmic tail. **a**) AFP-B7-38 and AFP-B7-5 are chimeric AFP proteins with a full-length or a truncated B7 cytoplasmic domain that retained 5 amino acids, respectively. **b**) 3T3 cells transiently transfected with plasmids coding for AFP-B7-38 or AFP-B7-5 were immunofluorescence stained for the HA epitope tag present on AFP and analyzed on a flow cytometer.

Membrane-tethered single-chain antibodies are susceptible to proteolytic cleavage from the plasma membrane, limiting accumulation of antibody on cells [57]. We choose to use human alpha fetal protein (AFP) (Figure 2a) as a model chimeric protein for further studies because it is resistant to proteolytic cleavage [57], allowing accumulation of high levels of both AFP-B7-38 and AFP-B7-5 on cells (Figure 2b). AFP is also a good reporter protein because it is monomeric protein and possesses a single N-linked glycosylation site that can be used to monitor its exit from the ER [58].

**Figure 3:**
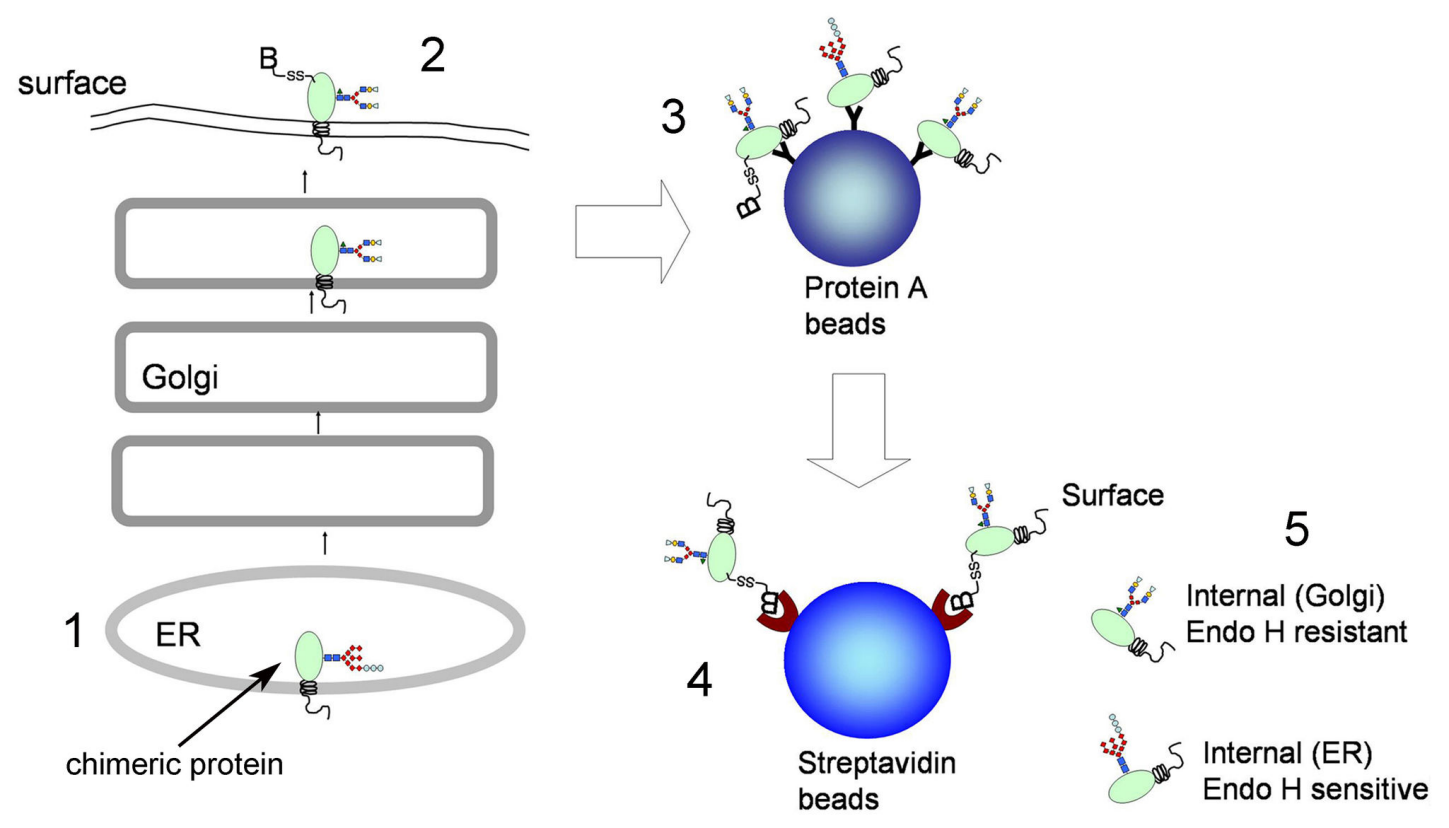
Outline of the pulse-chase procedure. **1**. Cells expressing AFP chimeric proteins were labeled with ^35^S-methionine and then chased with cold methionine for defined times. **2**. Surface proteins on viable cells were biotinylated (B) before both surface and intracellular AFP chimeric proteins are solubilized in detergent. **3**. Total intracellular and membrane AFP chimeric proteins are immunoprecipitated from cell lysates and collected on Protein A beads. **4**. After elution of total AFP chimeric proteins from protein A beads, the biotinylated (surface) AFP chimeric proteins are collected on streptavidin beads while intracellular AFP remained in the supernatant. **5**. ER and Golgi resident AFP can be differentiated by the presence of a single Endo H resident (Golgi form) or Endo H sensitive (ER form) oligosaccharide.

**Figure 4:**
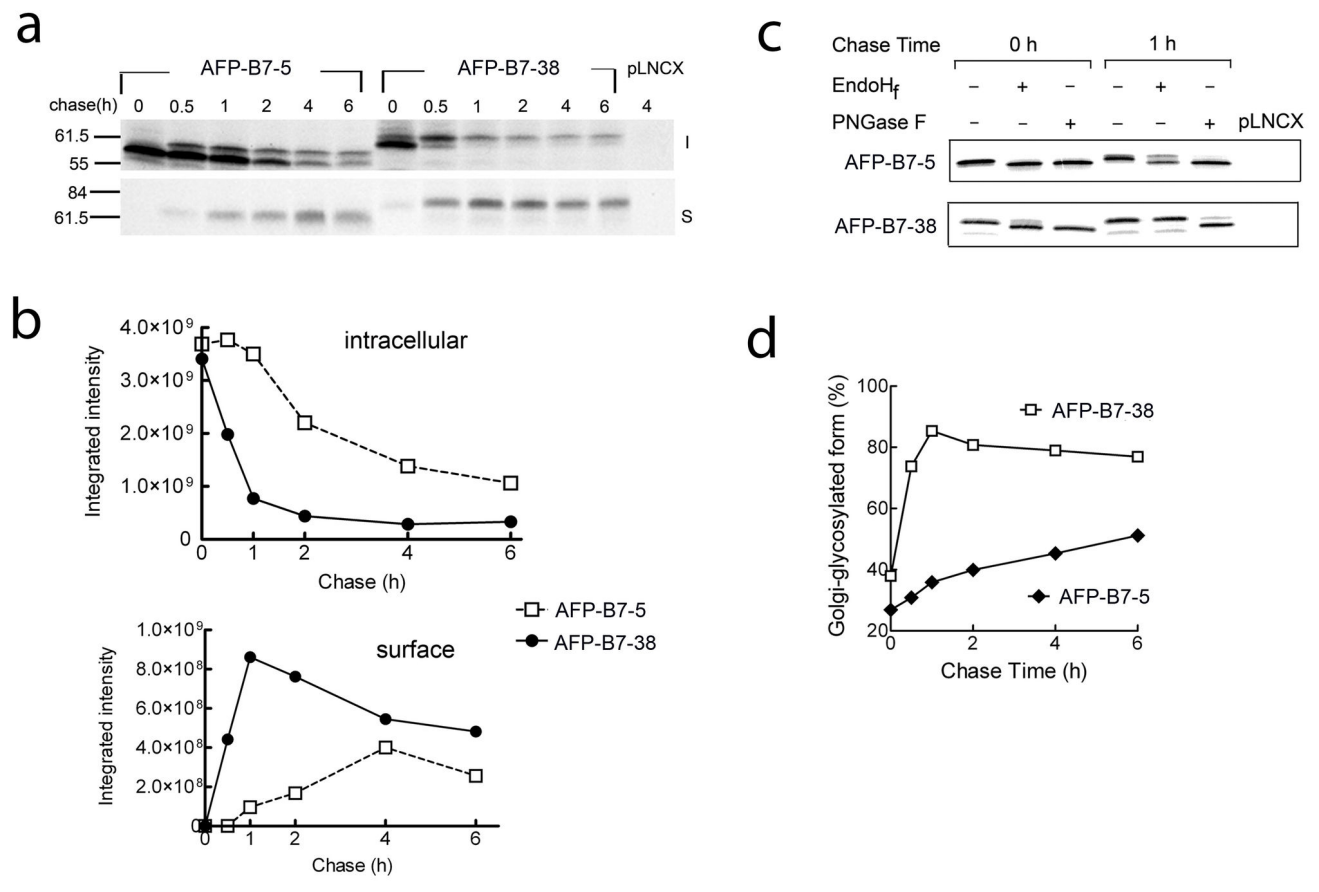
AFP intracellular transport can be followed by AFP glycosylation status. **a**) 3T3 cells stably expressing AFP-B7-5 or AFP-B7-38 were pulsed with ^35^S-methionine and chased with cold methionine for the indicated times. Surface (S, lower panel) or intracellular (I, upper panel) AFP at each time is shown. Vector-transfected control 3T3 cells are indicated as pLNCX. **b**) Protein bands in **panel a** were quantified to show the temporal change in ^35^S-methionine labeled intracellular (upper panel, both upper and lower bands) and surface (lower panel) AFP chimeric proteins. **c**) 3T3 cells stably expressing AFP-B7-5 or AFP-B7-38 were pulsed with ^35^S-methionine and then chased with cold methionine for 0 or 1 h. AFP immunoprecipitates were untreated or treated with EndoHf or PNGaseF deglycosylase as indicated. **d**) Quantification of the percentage of intracellular AFP chimera with complex-type N-linked carbohydrate (Golgi-glycosylated form) in relation to total intracellular AFP chimeric protein in ^35^S-methionine labeled cells at the indicated chase times.

The intracellular transport rate of AFP chimeric proteins was measured by pulse-chase analysis (Figure 3). 3T3 cells that stably expressed AFP-B7-38 or AFP-B7-5 were pulsed with ^35^S-methionine and chased in cold medium for defined times. Solubilized cell lysates were immunoprecipitated with anti-AFP antibody and separated into surface and intracellular fractions. AFP-B7-38 was rapidly transported to the cell surface as shown by reduced intracellular levels (upper panel) and a strong surface signal within 30 min (lower panel) (Figure 4a). By contrast, AFP-B7-5 reached the cell surface at a slower rate with the majority present in the intracellular fraction even after 2 h (Figure 4a). Quantification of the intracellular and surface band intensities confirmed more rapid transport of AFP-B7-38 as compared to AFP-B7-5 (Figure 4b).

To investigate if the two distinct sizes of AFP observed in the gels correspond to differences in the single N-linked oligosaccharide chain, AFP immunoprecipitates were treated with EndoH_f_, which can hydrolyze simple carbohydrates present in the ER or N-glycosidase F (PNGaseF), which can liberate both simple and complex N-linked oligosaccharides found in the Golgi [59]. At the end of ^35^S-methionine pulsing (0 h), AFP-B7-5 was fully digested by both EndoH_f_ and PNGase F, indicating an ER localization (Figure 4c). By contrast, a small portion of AFP-B7-38 was resistant to EndoH_f_, indicating that some AFP-B7-38 had already entered the Golgi (Figure 4c). After chasing 1 h, the majority of AFP-B7-5 remained sensitive to EndoH_f_, indicating retention in the ER, whereas the majority of AFP-B7-38 was EndoH_f_ resistant, indicating passage of AFP-B7-38 to the medial Golgi. Quantification of the amount of EndoH_f_-resistant AFP as a percentage of total intracellular AFP clearly demonstrated that AFP-B7-38 exited the ER more rapidly than AFP-B7-5 (Figure 4d).

**Figure 5:**
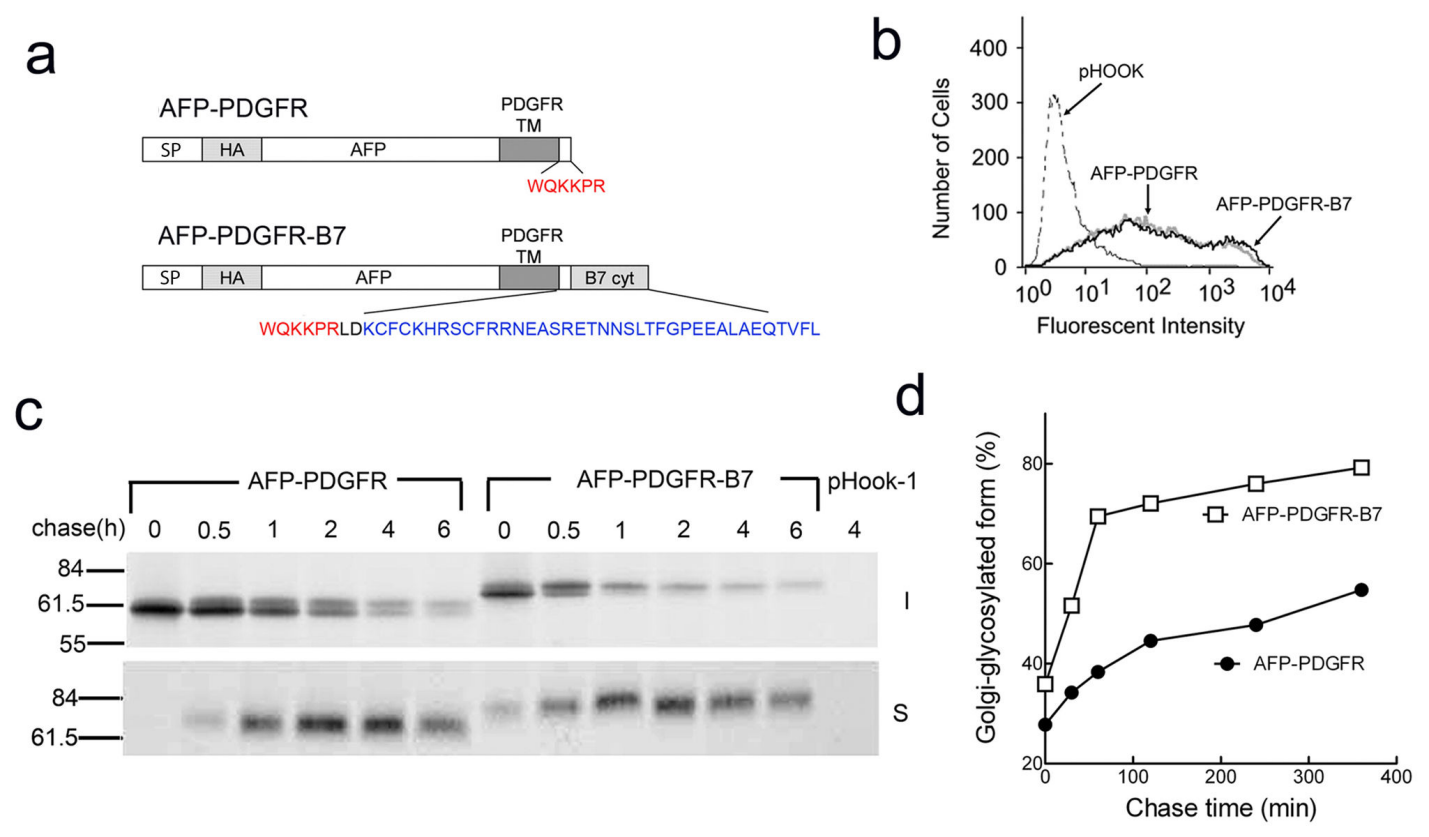
The B7 cytoplasmic domain can accelerate the intracellular transport of a chimeric AFP protein. **a**) AFP-PDGFR-B7 has the B7 cytoplasmic domain appended to AFP-PDGFR, in which AFP replaces the scFv in scFv-PDGFR (Figure 1c). **b**) 3T3 fibroblasts that were transiently-transfected with plasmids coding for AFP-PDGFR or AFP-PDGFR-B7 were immunofluorescence stained for the HA epitope tag on AFP. **c**) 3T3 cells expressing AFP-PDGFR or AFP-PDGFR-B7 were pulse-chased for the indicated times before surface (S, lower panel) and intracellular (I, upper panel) AFP chimeric proteins were immunoprecipitated, separated by SDS PAGE and visualized by autoradiography. Vector-transfected control cells are indicated as pHook-1. **d**) The percentage of intracellular Golgi-glycosylated AFP chimera in relation to total intracellular AFP chimeric protein in ^35^S-methionine labeled cells at the indicated chase times are shown.

To further examine if the B7 cytoplasmic tail could accelerate the intracellular transport rate of AFP, we created a chimera in which the B7 cytoplasmic tail was appended to AFP-PDGFR (Figure 5a). Both AFP-PDGFR and AFP-PDGFR-B7 were expressed at similar levels on the surface of transiently-transfected 3T3 cells (Figure 5b). However, AFP-PDGFR-B7 was transported more rapidly to the surface of 3T3 cells as shown both by pulse-chase (Figure 5c) and measurement of the amount of intracellular Golgi-glycosylated (complex carbohydrate) AFP (Figure 5d). In subsequent studies, we used the percentage of intracellular Golgi-glycosylated AFP compared to total intracellular AFP as a measure of intracellular transport rate since this value is self-normalized and is insensitive to gel loading and membrane transfer variations. We conclude that 1) the commonly used measurement of surface expression is a poor indicator of intracellular transport rate for proteins that are stably expressed on the cell surface, 2) the B7 cytoplasmic domain can enhance the intracellular transport of both a single-chain antibody and a reporter protein (AFP), and 3) this effect is independent of the transmembrane domain.

### Chimeric proteins possessing the B7 cytoplasmic domain are transported via the conventional COPII pathway

To investigate if export of chimeric proteins possessing the B7 cytoplasmic domain from the ER occurs via classical coatomer complex II (COPII) mediated transport, we examined the effects of dominant-negative mutants of Sar1 and Rab1 on protein transport. The GTPase Sar1 is required for COPII assembly and fusion [60]. The constitutively trans-dominant inactive Sar1 mutant (Sar1[T39N]) blocks COPII vesicle formation [60] and the dominant-negative constitutively active GTP-restricted SAR1 mutant Sar1[H79G] causes COPII cargos to accumulate in pre-budding complexes [49]. The GTPase Rab1 regulates conventional anterograde COPII-mediated transport between the ER and Golgi whereas Rab1DN, which possesses a N124I mutation, interferes with the conventional transport pathway [61].

**Figure 6:**
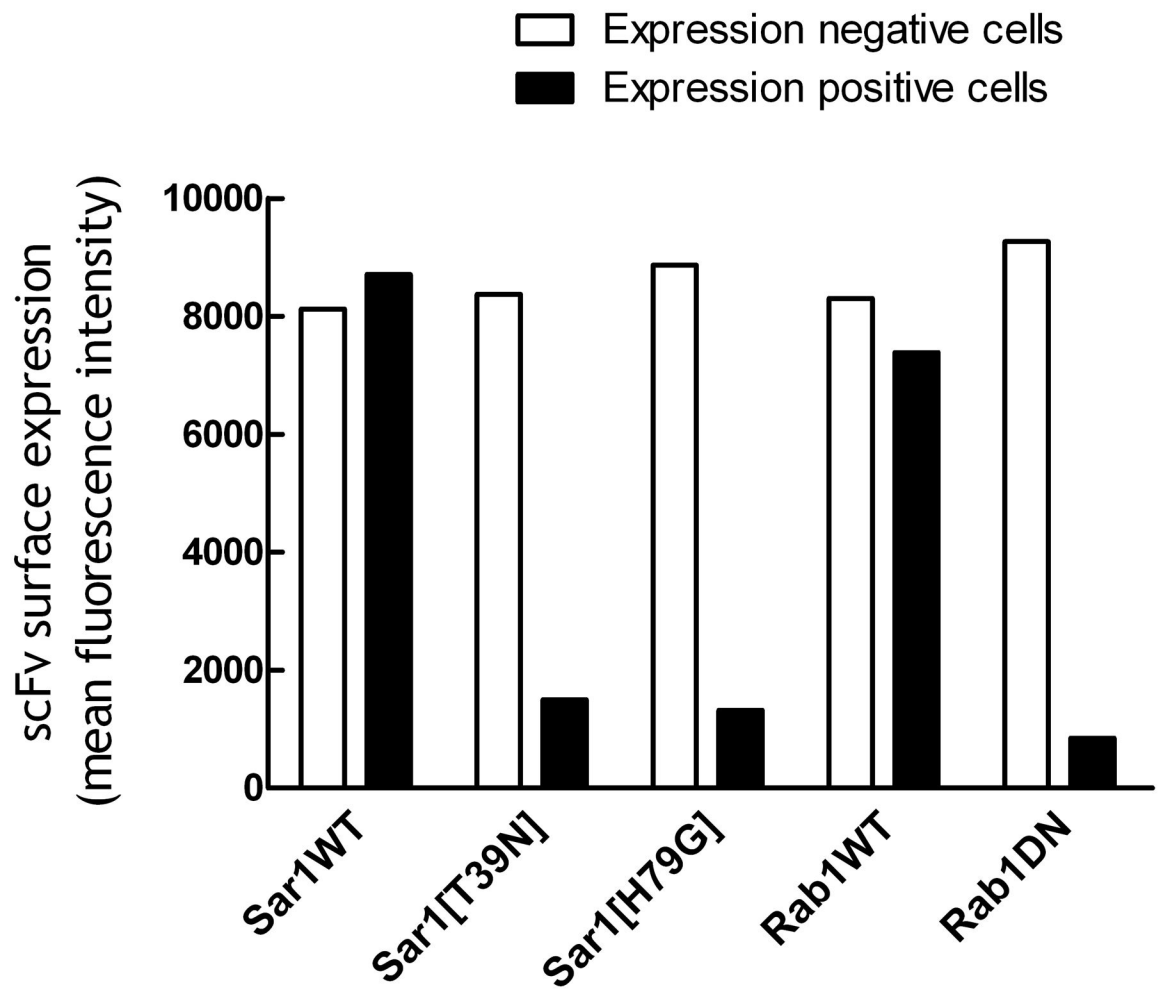
Transport of B7 chimeric proteins depends on Sar1 and Rab1 GTPases. 3T3 cells that stably expressed scFv-B7-38 were transiently transfected with eGFP-Sar1WT, mCherry-Sar1[T39N], eGFP-Sar1[H79G], eGFP-Rab1WT or mCherry-Rab1DN plasmids. Forty-eight hours later, the cells were immunofluorescence stained with anti-HA antibody to detect the levels of HA-tagged scFv-B7-38 on the surface of the cells. Results show the mean surface immunofluorescence levels of scFv-B7-38 on live cells that were successfully transfected and expressed eGFP or mCherry (expression positive cells, filled bars) compared to live cells that were not transfected and thus did not express eGFP or mCherry (expression negative cells, open bars).

We transiently transfected Sar1 and Rab1 genes into 3T3 cells that stably expressed scFv-B7-38 (a membrane-anchored single-chain antibody) and then measured the accumulation of single-chain antibody on the surface of the cells as a readout of scFv-B7-38 intracellular transport rate (Figure 1). The surface expression of scFv on the population of scFv-B7-38 cells that were successfully transfected with eGFP-Sar1WT, mCherry-Sar1[T39N], eGFP-Sar1[H79G], eGFP-Rab1WT or mCherry-Rab1DN and therefore expressed eGFP or mCherry fluorescence was compared with the population of scFv-B7-38 cells that were not successfully transfected and thus did not express these fluorescence fusion proteins. Expression of eGFP-Sar1WT did not affect the levels of scFv-B7-38 on cells whereas surface expression of scFv-B7-38 was decreased by about 82% to 85% in cells that expressed mCherry-Sar1[T39N] or eGFP-Sar1[H79G] as compared to their untransfected counterparts (Figure 6). Likewise, expression of mCherry-Rab1DN decreased the expression of scFv-B7-38 on transfected cells by 91% (Figure 6). We conclude that chimeric proteins possessing the B7 cytoplasmic domain are exported from the ER via classical coatomer complex II (COPII) mediated transport.

### Progressive deletion of the B7 cytoplasmic domain reduces the rate of intracellular transport

**Figure 7:**
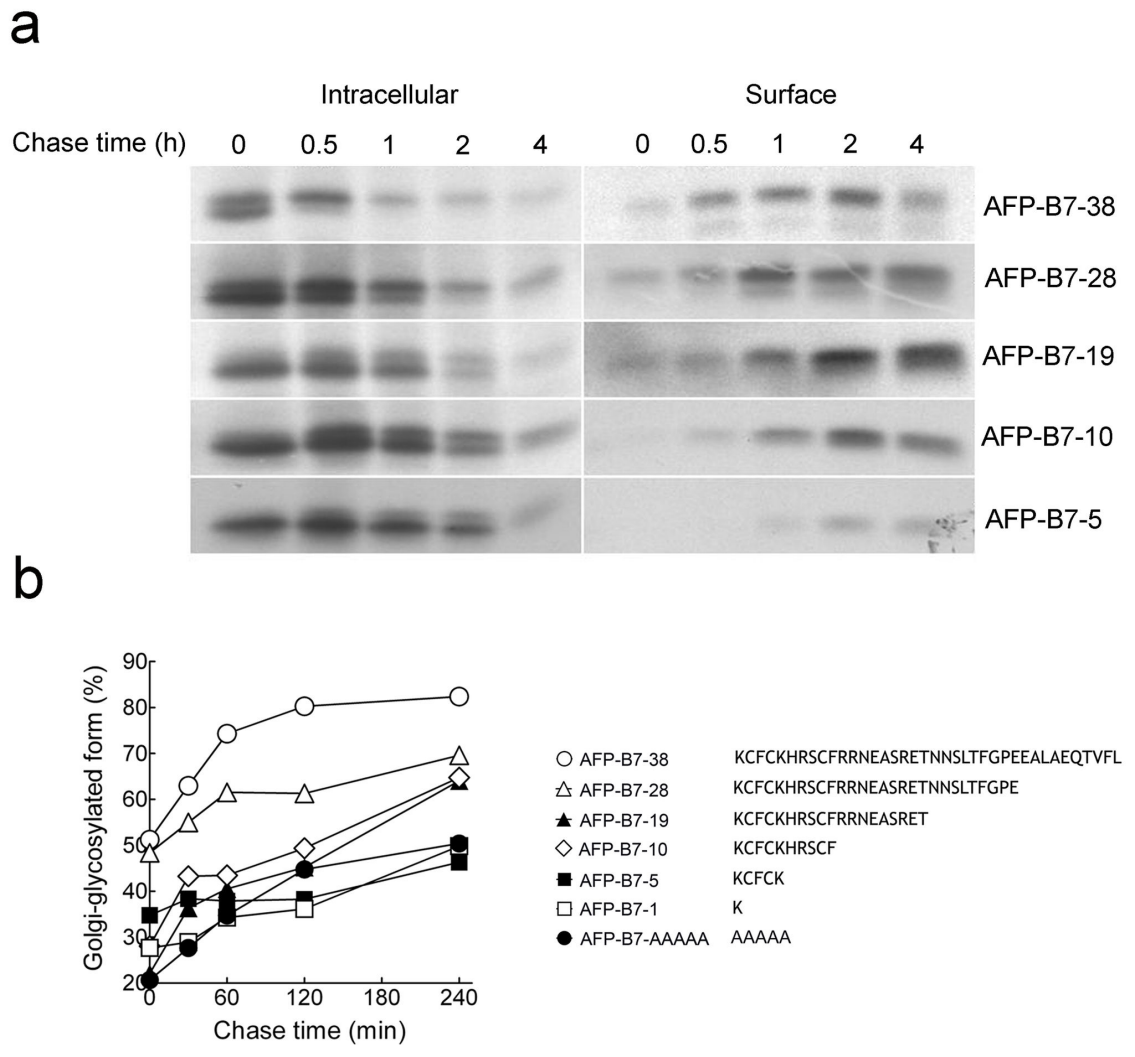
Intracellular transport rates of AFP chimeric proteins with truncated cytoplasmic domains. **a**) 3T3 cells expressing AFP-B7-38 or truncation variants were pulse-chased for the indicated times before intracellular (left panel) or surface (right panel) AFP chimeric proteins were immunoprecipitated, separated by SDS PAGE and visualized by autoradiography. **b**) The percentage of intracellular Golgi-glycosylated AFP relative to total intracellular AFP for cytoplasmic domain truncation variants is shown.

Several specific amino acid motifs in the cytoplasmic domain of membrane proteins have been identified that can enhance their ER export [26,30,31,62-73]. To help identify putative ER export motifs, we deleted progressively greater portions of the B7 cytoplasmic domain and measured intracellular transport rates. Sequential removal of amino acids resulted in progressively slower intracellular transport as seen in both delayed accumulation of AFP on the cell surface and transport from the ER to the Golgi as assessed by attachment of Endo Hf-resistant carbohydrate on AFP (Figure 7a). Quantification of the amount of intracellular AFP with complex carbohydrate confirmed progressively slower intracellular transport of the chimeric proteins as more of the B7 cytoplasmic tail was removed (Figure 7b). These results are consistent with enhancement of intracellular transport by the B7 cytoplasmic domain, but no specific section of the B7 cytoplasmic tail appeared to promote ER export.

To rule out the possibility that deletion of C-terminal amino acids resulted in progressive exposure of a cryptic ER retention signal in the remaining amino acids present in the deletion mutants, we replaced the juxtamembrane five amino acids in AFP-B7-5 with alanine resudues to form AFP-B7-AAAAA. However, AFP-B7-AAAA was also transported slowly (Figure 7b), demonstrating that a cryptic ER retention signal was not present in the short cytoplasmic tail of AFP-B7-5.

### A minimal cytoplasmic tail is required for stable membrane integration

**Figure 8:**
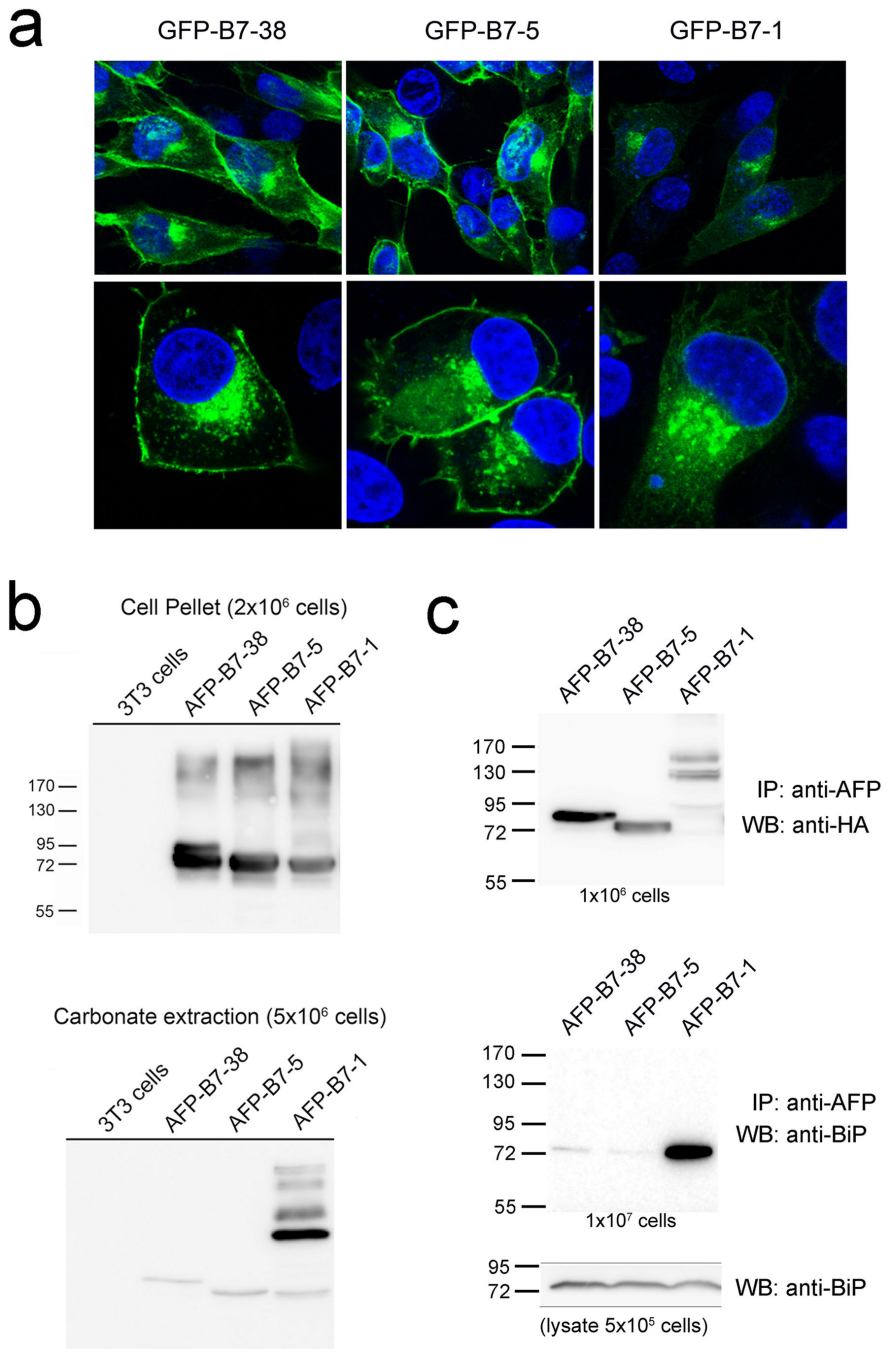
A minimum cytoplasmic tail is required for stable membrane association. **a**) 3T3 cells transiently expressing GFP-B7-38, GFP-B7-5 or GFP-B7-1 were stained with DAPI (blue) and then observed under a fluorescence microscope. The lower panels show a higher magnification view. **b**) 10^7^ 3T3 cells or stable 3T3 transfectants expressing AFP-B7-38, AFP-B7-5 or AFP-B7-1 were lysed in 200 µl carbonate buffer and centrifuged at 100,000xg. The insoluble fraction from 2x10^6^ cells (upper panel) or carbonate soluble fraction from 5x10^6^ cells (lower panel) were separated by SDS PAGE and then immunoblotted with anti-HA antibody to visualize AFP chimeric proteins. **c**) 3T3 cells stably expressing AFP-B7-38, AFP-B7-5 or AFP-B7-1 were solubilized and immunoprecipitated with anti-AFP antibody. One tenth of the mixture, corresponding to 10^6^ cells, was separated by SDS PAGE and immunoblotted with anti-HA antibody to visualize AFP chimeric proteins (upper panel). The remaining lysate was separated by SDS PAGE and then immunoblotted with rabbit anti-BiP (GRP78) antibody (middle panel). The lower panel shows an immunoblot for total BiP in whole cell lysates from 5x10^5^ cells.

We wished to modify the B7 cytoplasmic tail to help identify potential ER export motifs. Both AFP-B7-1, which retained a single cytoplasmic tail amino acid, and AFP-B7-5, which retained five cytoplasmic tail amino acids, displayed similar slow intracellular transport rates (Figure 7b) and thus appeared to be suitable candidates for a minimal cytoplasmic tail for further investigations. A minimum number of cytoplasmic amino acids, however, is often required to allow stable retention and proper orientation of transmembrane proteins in membranes as typified by the “positive inside” rule, in which arginine and lysine residues are often found on the juxtamembrane region of the cytoplasmic side of transmembrane proteins [74,75]. To verify that these minimal tails were sufficient for proper orientation of chimeric proteins, we first replaced AFP with GFP to visualize the location of selected chimeras in cells. GFP-B7-38 and GFP-B7-5 displayed a similar localization pattern with obvious accumulation on the plasma membrane, but GFP-B7-1 was predominately retained intracellularly (Figure 8a).

We further treated stable cell transfectants with sodium carbonate, which is routinely used to determine if proteins are firmly attached to membranes [76,77]. Immunoblotting of the carbonate soluble fraction revealed elevated levels of AFP-B7-1 as compared to AFP-B7-38 and AFP-B7-5 (Figure 8b, lower panel), indicating a portion of AFP-B7-1 was not tightly associated with cell membranes. The molecular size of the carbonate soluble fraction of AFP-B7-1 was also larger than expected, indicating formation of higher order aggregates of AFP-B7-1 that could not be dissociated by boiling in SDS PAGE buffer.

We further examined if chimeric proteins interacted with BiP, a major ER resident chaperone that can recognize exposed hydrophobic regions of nascent proteins [78,79]. Lysates prepared from stable cell transfectants were immunoprecipitated with anti-AFP antibodies, electrophoresed, transferred to nitrocellulose membranes and then probed with anti-AFP (Figure 8c, upper panel) or anti-BiP antibodies (Figure 8c, middle panel). BiP was clearly co-immunoprecipitated with AFP-B7-1. We conclude that the short cytoplasmic tail present in AFP-B7-1 is insufficient for stable membrane integration.

**Figure 9:**
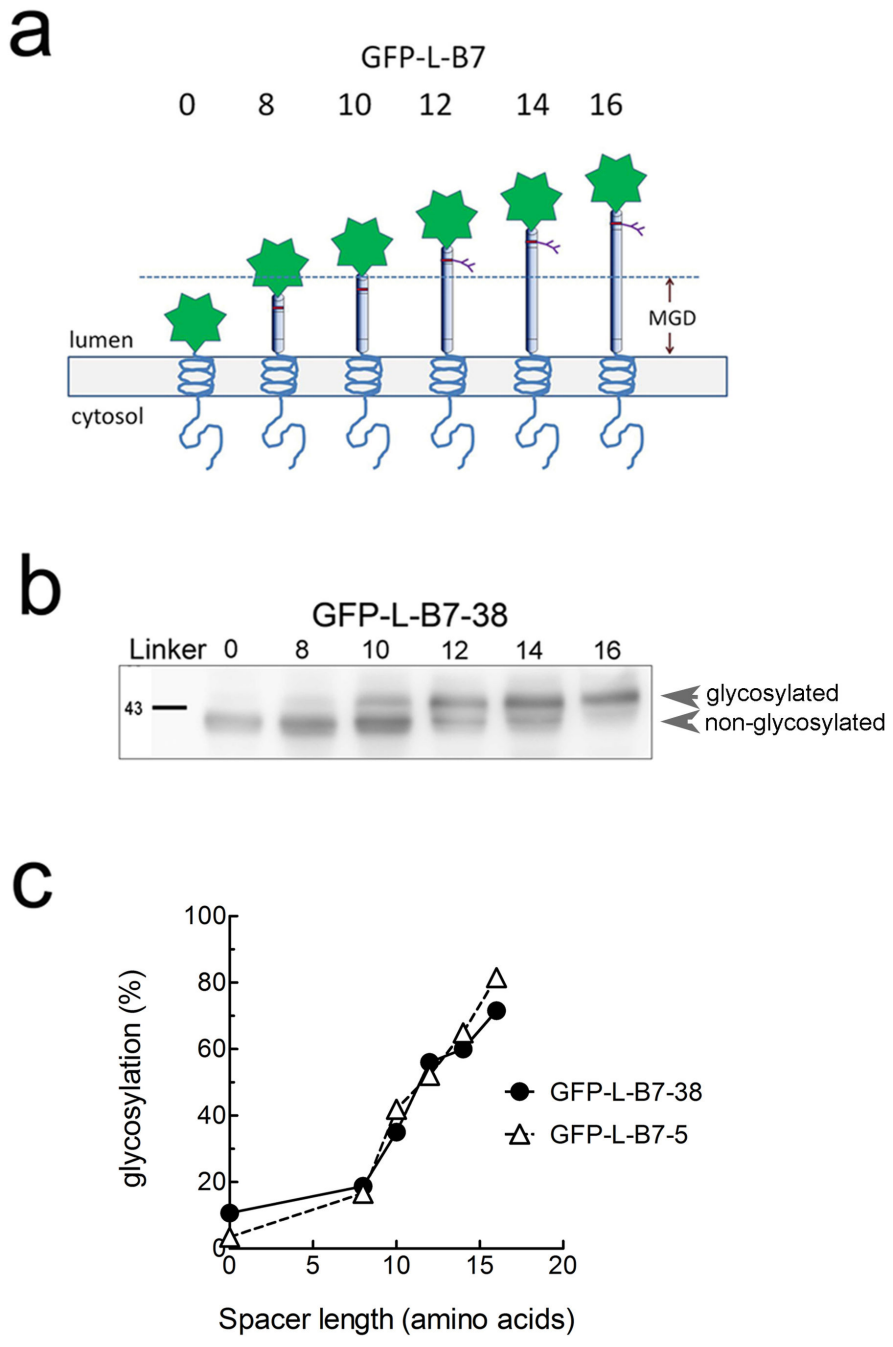
N-glycosylation mapping of chimeric proteins. **a**) A series of GFP chimeric proteins with a full length cytoplasmic tail (GFP-L-B7-38) or a truncated cytoplasmic tail (GFP-L-B7-5) were generated with a single N-linked glycosylation sites engineered at variable distances (indicated by the number of amino acids in the linker domain, L) from the ER luminal membrane. Accessibility of enzymes responsible for glycosylation provides a measure the distance of the glycosylation site from the ER lumen membrane, and indirectly provides a measure of the position of the transmembrane domain in the ER membrane. **b**) 3T3 cells were transiently transfected with GFP-0-B7-38, GFP-8-B7-38, GFP-10-B7-38, GFP-12-B7-38, GFP-14-B7-38 or GFP-16-B7-38, harvested after 48 h, boiled in SDS PAGE buffer, separated on a SDS PAGE and immunoblotted with anti-HA antibody to visualize GFP chimeric proteins. Results show the glycosylated (upper band) versus non-glycosylated (lower band) forms of GFP-L-B7-38 with the indicated number of amino acids between the TM and N-liked glycosylation site. **c**) The amount of chimeric protein that is glycosylated as a percentage of total chimeric protein is shown versus spacer length (number of amino acids between the TM and glycosylation site) for GFP chimeric proteins with the original B7 cytoplasmic domain (GFP-L-B7-38) or a truncated B7 cytoplasmic domain (GFP-L-B7-5). Both chimeric proteins displayed similar dependence on linker length for glycosylation, indicating similar orientation and position of their transmembrane domains in the ER lumen.

We employed glycosylation mapping to further verify that the 5 amino acid cytoplasmic tail in AFP-B7-5 was properly positioned in the ER membrane. N-glycosylation mapping, in which one measures the amount of glycosylation at an engineered glycosylation site placed at defined distances from the ER membrane, can accurately determine the position of a transmembrane helix in the ER membrane because oligosaccharyltransferase, which catalyzed N-glycosylation, is sterically-hindered by close proximity to the ER lumen membrane [80,81]. Efficient addition of N-linked oligosaccharides therefore requires a minimal distance from the ER membrane, typically corresponding to a distance of 12-14 amino acids [80]. We created a series of chimeric proteins in which GFP was linked a spacer containing a single N-linked glycosylation site followed by defined numbers of amino acids to the B7 transmembrane and full cytoplasmic tail (GFP-L-B7-38) or truncated cytoplasmic tail (GFP-L-B7-5) (Figure 9a). The percentage of chimeric protein that was glycosylated clearly depended on the distance of the N-linked glycosylation site from the ER membrane (Figure 9b). Quantification of the degree of chimeric protein glycosylation showed that both GFP-L-B7-5 and GFP-L-B7-37 required spacers equivalent to about 11 amino acids to achieve 50% glycosylation (Figure 9c), demonstrating that the short cytoplasmic tail in GFP-L-B7-5 was sufficient for stable integration of chimeric proteins in cell membranes and therefore represents a suitable basis for construction of cytoplasmic tail mutants. All subsequent cytoplasmic tail mutants therefore retained the juxtamembrane five amino acids present in AFP-B7-5 to ensure proper membrane integration.

### The B7 cytoplasmic tail does not contain a linear ER export motif

**Figure 10:**
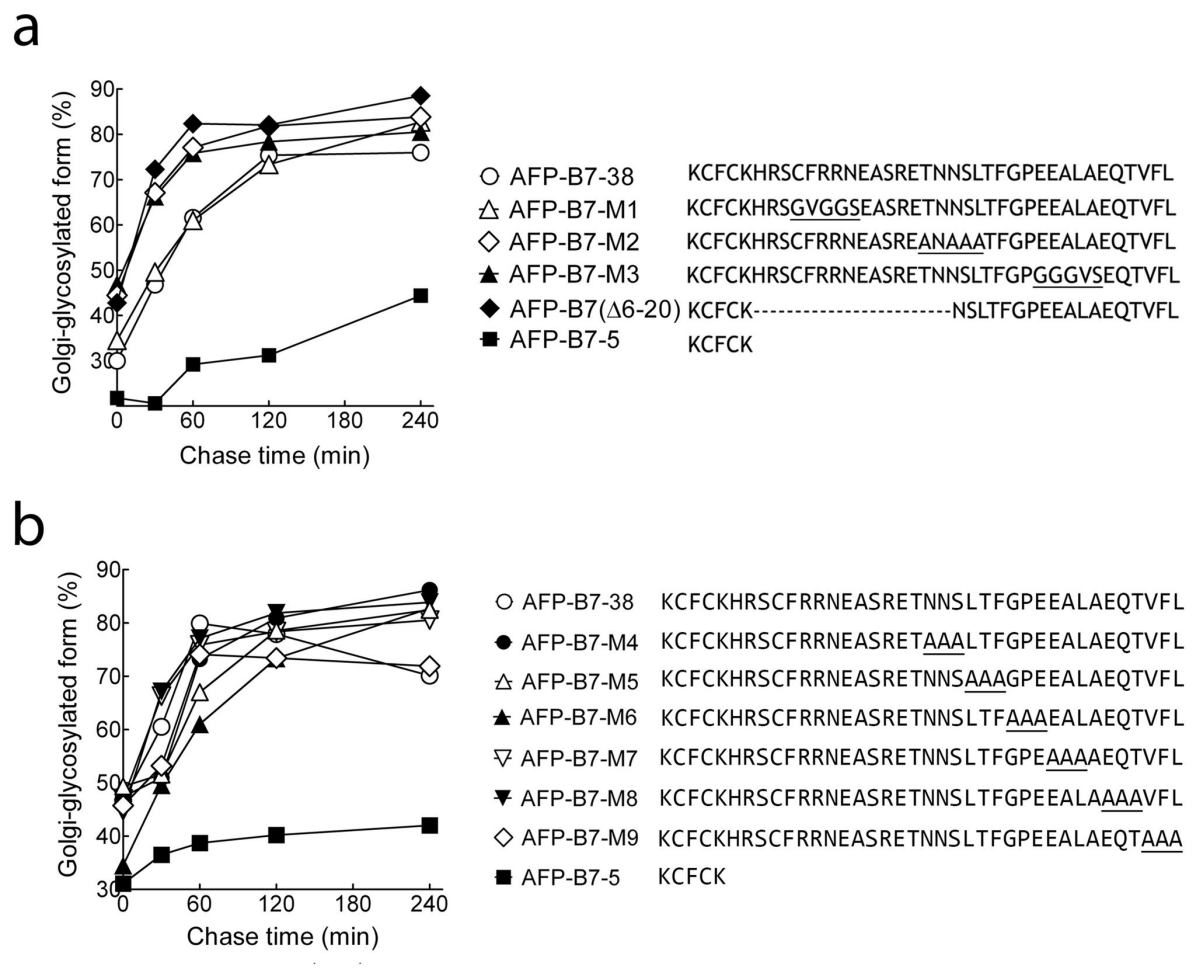
The B7 cytoplasmic tail does not appear to possess a linear ER export motif. **a**) The intracellular transport, as measured by the ratio of intracellular Golgi-glycosylated AFP to total intracellular AFP, of chimeric proteins with mutations in putative ER export motifs (underlined) or a deletion of amino acids 6 to 20 (AFP-B7(Δ6-20)) are shown. **b**) Alanine scanning analysis was performed over amino acids 20-38 in the B7 cytoplasmic domain. The intracellular transport rate, quantified as the percentage of intracellular Golgi-glycosylated AFP relative to total intracellular AFP, is shown.

We mutated three cytoplasmic regions similar to previously determined ER export motifs to investigate if intracellular transport rate would be affected [26,30,31,62-73]. AFP-B7-M1, in which ^9^CFRRN^13^ was changed to^9^GVGGS^13^, was transported to the Golgi as rapidly as AFP-B7-38 (Figure 10a). Likewise, AFP-B7-M2 (^19^TNNSL^23^ →^19^ANAAA^23^) and AFP-B7-M3 (^28^EEALA^32^ →^28^GGGVS^32^) mutants were rapidly transported to the Golgi (Figure 10a). Deletion of cytoplasmic amino acids 6 to 20 in AFP-B7(Δ6-20) did not reduce transport (Figure 10a), showing this portion of the cytoplasmic tail cannot contain an important ER export signal. Alanine scanning of the remaining amino acids (20 to 38) did not decrease protein transport to the Golgi (Figure 10b), indicating that the B7 cytoplasmic tail does not contain a linear ER export motif.

**Figure 11:**
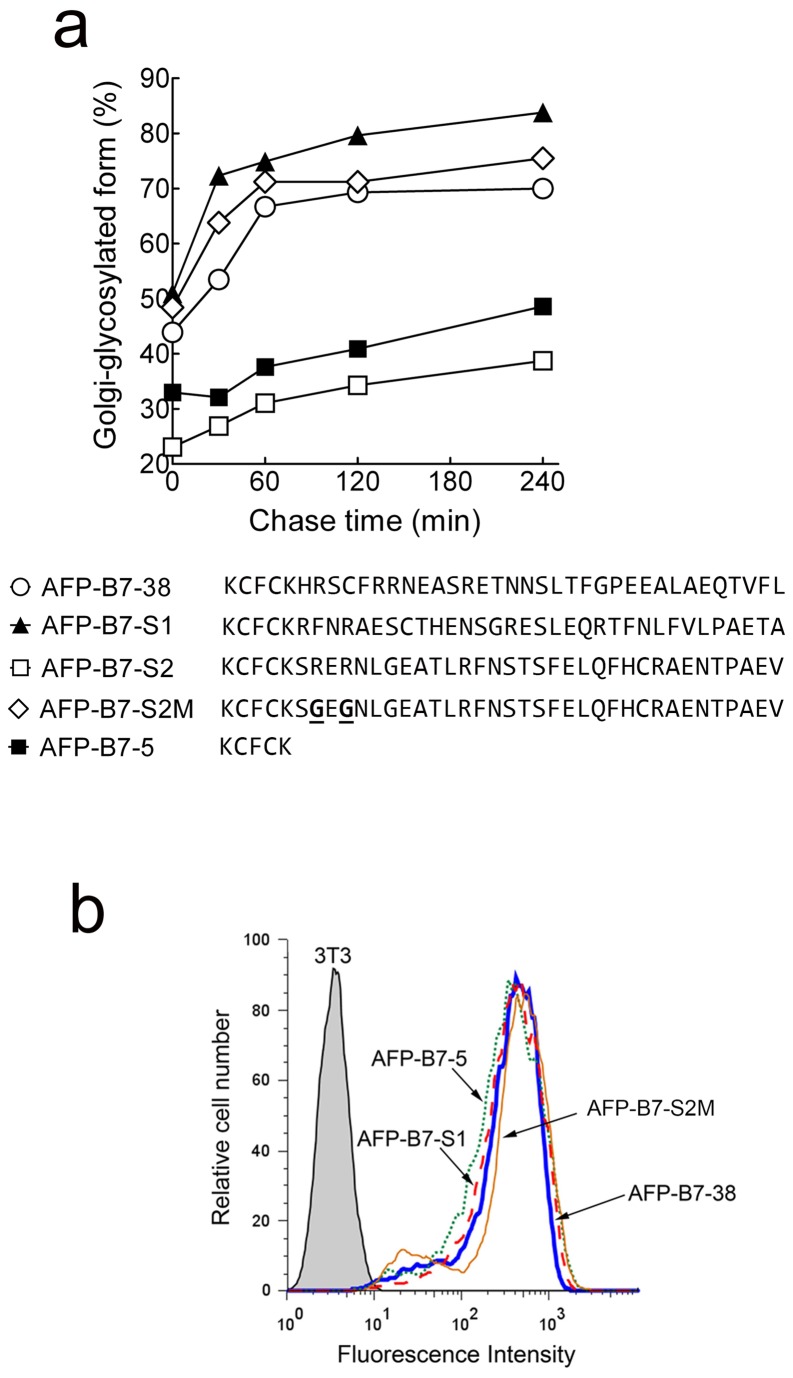
Scrambling the position of amino acids in the B7 cytoplasmic tail does not hinder intracellular transport. **a**) The positions of amino acids in the B7 cytoplasmic tail were scrambled in AFP-B7-S1 and AFP-B7-S2. A putative RXR ER retention motif in the S2 cytoplasmic tail was removed by changing two arginine residues to glycine residues (underlined) to form AFP-B7-S2M. The intracellular transport rate, quantified as the percentage of intracellular Golgi-glycosylated AFP relative to total intracellular AFP, is shown. **b**) The expression of AFP-B7-38, AFP-B7-S1, AFP-B7-S2M and AFP-B7-5 on the surface of stably transfected 3T3 cells was determined by flow cytometry.

To further rule out the presence of an ER export motif in the B7 cytoplasmic tail, we generated synthetic cytoplasmic tails using the identical amino acids present in AFP-B7-38 but in a scrambled order to produce AFP-B7-S1 and AFP-B7-S2. Pulse-chase analysis showed that AFP-B7-S1 rapidly reached the Golgi whereas AFP-B7-S2 was transported even more slowly that AFP-B7-5 (Figure 11a). Examination of the AFP-B7-S2 cytoplasmic tail, however, revealed that we inadvertently generated a RER motif, similar to previously reported RXR motifs employed for ER retrieval of resident ER proteins by direct interaction with the COPI coat [82-86]. We therefore eliminated the RxR motif by replacement of the two arginine residues with glycine residues to generate AFP-B7-S2M. Indeed, AFP-B7-S2M was rapidly transported to the Golgi as determined by pulse-chase analysis (Figure 11a). Both AFP-B7-S1 and AFP-B7-S2M were highly expressed on the surface of stable 3T3 transfectants (Figure 11b). Taken together, we conclude that the B7 cytoplasmic tail does not possess a linear ER export motif.

### Many reported ER export motifs are rarely found in the cytoplasmic tails of type I membrane proteins

The apparent lack of a linear export motif in the B7 cytoplasmic tail prompted us to examine how often previously reported ER export motifs are found in type I transmembrane proteins. Toward this end, we examined the frequency of several reported motifs in the cytoplasmic domains of 984 human and 782 mouse type I transmembrane proteins. The diacidic motif (D/ExD/E), which interacts with the B-site of Sec24p [25], is the most common ER export motif in the cytoplasmic domain of type I membrane proteins, occurring 2295 times in 984 human transmembrane proteins and 1858 times in 782 mouse transmembrane proteins (Table 1). The DxD and ExE motifs occur at 20-25% greater frequencies than expected if these amino acids occurred randomly, consistent with an important role for diacidic motifs in ER exit. However, 337 of 984 human and 259 of 782 mouse type I transmembrane proteins did not contain a diacidic motif and it is unclear how many of the remaining diacidic motifs functionally promote ER export.

**Table 1:** Frequency of putative acidic ER export motifs in the cytoplasmic tails of type I transmembrane proteins.

**Motif**	**Proteins**	**Human**			**Mouse**			**Citations**
		**Expected occurrences**	**Actual occurrences**	**% difference**	**Expected occurrences**	**Actual occurrences**	**% difference**	
DxE	VSV G protein, sys1p, GONST1, CASP	562	572	1.8	456	453	-0.7	[30,62-64]
DxD	Cystic fibrosis transmembrane conductance regulator	422	531	25.8	348	446	28.2	[102]
ExE	TASK-3 acid-sensitive potassium channel, angiotensin II receptor	749	888	18.6	598	732	22.4	[34,103]
ExD	angiotensin II receptor	562	576	2.5	456	471	3.3	[34]
D/E-x-D/E	Kir inwardly rectifying potassium channels	2295	2567	11.9	1858	2102	13.1	[29,89]
FxYENE	Kir inward rectifier K. channels	0	0	0	0	0	0	[29,89]

The expected random number of occurrences of each motif in the cytoplasmic tails of 984 human or 782 mouse non-redundant type I transmembrane proteins is compared with the actual number of occurrences of the motif. The % difference was calculated as % 100 * (actual occurrences – expected occurrences / expected occurrences).

An aromatic motif (FF, YY, FY, YF), which can be bound by the COPII components Sec 23p and Sec24p [31], is commonly found in transmembrane proteins but at 7 to 25% lower frequencies than randomly expected (Table 2). On the other hand, a valine residue at the very C-terminal position of the cytoplasmic tail of transmembrane proteins, reported to function as a forward transport signal by binding to specific Sec24p isoforms [31,33] or to the Golgi matrix proteins GRASP65 and GRASP55 [87], is highly over represented with 145 of 984 human transmembrane proteins and 114 of 782 mouse transmembrane proteins possessing a terminal valine, representing frequencies about 170% greater than randomly expected (Table 2). However, the presence of a C-terminal valine does not guarantee rapid ER egress. For example, HLA-A forward transport was unaffected by deletion of a naturally-occurring C-terminal valine [88]. Terminal I, A, II and LL motifs were also highly over represented in type I TM proteins.

**Table 2:** Frequency of putative hydrophobic and aromatic ER export motifs in the cytoplasmic tails of type I transmembrane proteins.

**Motif**	**Proteins**	**Human**			**Mouse**			**Citations**
		**Expected occurrences**	**Actual occurrences**	**% difference**	**Expected occurrences**	**Actual occurrences**	**% difference**	
RL	GABA transporter 1	800	763	-4.6	663	657	-0.9	[27]
Terminal LL	ERGIC-53	6.8	10	47	5.9	8	36	[31]
Terminal II	ERGIC-53	1.3	2	54	1.1	3	173	[31]
Terminal V	Stem cell factor, ERGIC-53, CD8α, Frizzled4, FXYD7, HLA-F	53	145	174	43	114	165	[31,33,87,88,104]
Terminal I		36	64	78	29	66	128	[31]
Terminal A		65	105	62	50	86	72	
Terminal L		82	102	24.4	68	76	11.8	
F at -2	ERGIC-53	28	30	7.1	23	26	13	[31]
Y at -2	ERGIC-53	31	40	29	25	28	12	[31]
FF	ERGIC-53, p24	119	101	-15	98	85	-13	[28,105-107]
YY	ERGIC-53	146	130	-11	124	107	-14	[31]
FY	Erv46p	132	104	-21	110	80	-27	[107]
YF		132	123	-7	110	101	-8	

The expected random number of occurrences of each motif in the cytoplasmic tails of 984 human or 782 mouse non-redundant type I transmembrane proteins is compared with the actual number of occurrences of the motif. The % difference was calculated as % 100 * (actual occurrences – expected occurrences / expected occurrences).

Several previously reported ER export motifs are rarely found in type I TM proteins. For example, although FxYENE was shown to mediate ER export of Kir potassium channels [29,89], this motif was not present in any human or mouse type I transmembrane protein (Table 3). Likewise, the MELADL motif present in loop 6 between transmembrane helices 6 and 7 of hamster Scap, a sterol sensor [70], the FNxxLLxxxL motif found in the CT of the seven transmembrane domain vasopressin V1b/V3 receptor [69], the YTDIEM motif in the CT of the vesicular stomatitis virus G protein [68], the HLFY motif present in the N-methyl-D-aspartic acid receptor [67] and the SWTY motif found by library screening are not found or are present in only a single type I TM protein, indicating that these are unlikely to be general ER export motifs [66].

**Table 3:** Frequency of putative ER export motifs in the cytoplasmic tails of type I transmembrane proteins.

**Motif**	**Proteins**	**Human**			**Mouse**			**Citations**
		**Expected occurrences**	**Actual occurrences**	**% difference**	**Expected occurrences**	**Actual occurrences**	**% difference**	
MELADL	Hamster Scap	0	0	0	0	0	0	[70]
NPF	Sed5p	12	6	-50	10	5	-50	[71]
Lxx-L/M-E	Sed5, Bet1	91	107	18	79	92	17	[72]
F(X)_6_LL	α_2B_-adrenergic receptor, angiotensin II type 1A receptor	29	19	-34.5	23	26	-11.5	[26,73]
FNxxLLxxxL	Vasopressin V1b/V3 receptor	0	0	0	0	0	0	[69]
YTDIEM	VSV G protein	0	0	0	0	0	0	[68]
HLFY	*N*-methyl-D-aspartic acid receptor	0.3	1	233	0.3	1	233	[67]
SWTY	Synthetic protein	0.3	0	-100	0.2	0	-100	[66]
VMI	GAT1	6	17	183	5	10	100	[65]

The expected random number of occurrences of each motif in the cytoplasmic tails of 984 human or 782 mouse non-redundant type I transmembrane proteins is compared with the actual number of occurrences of the motif. The % difference was calculated as % 100 * (actual occurrences – expected occurrences / expected occurrences).

These studies indicate that many previously reported linear ER export motifs are rarely found in the cytoplasmic tails of type I transmembrane proteins or are only found at the randomly expected frequencies. By contrast, acidic amino acids (DxD and ExE) and terminal hydrophobic amino acids (I, V, L, A) are found in the cytoplasmic tails of human and mouse type I transmembrane proteins at higher frequencies than expected by chance, supporting an important role for these residues in intracellular transport.

### The properties of specific cytoplasmic domain amino acids do not affect transport rate

**Figure 12:**
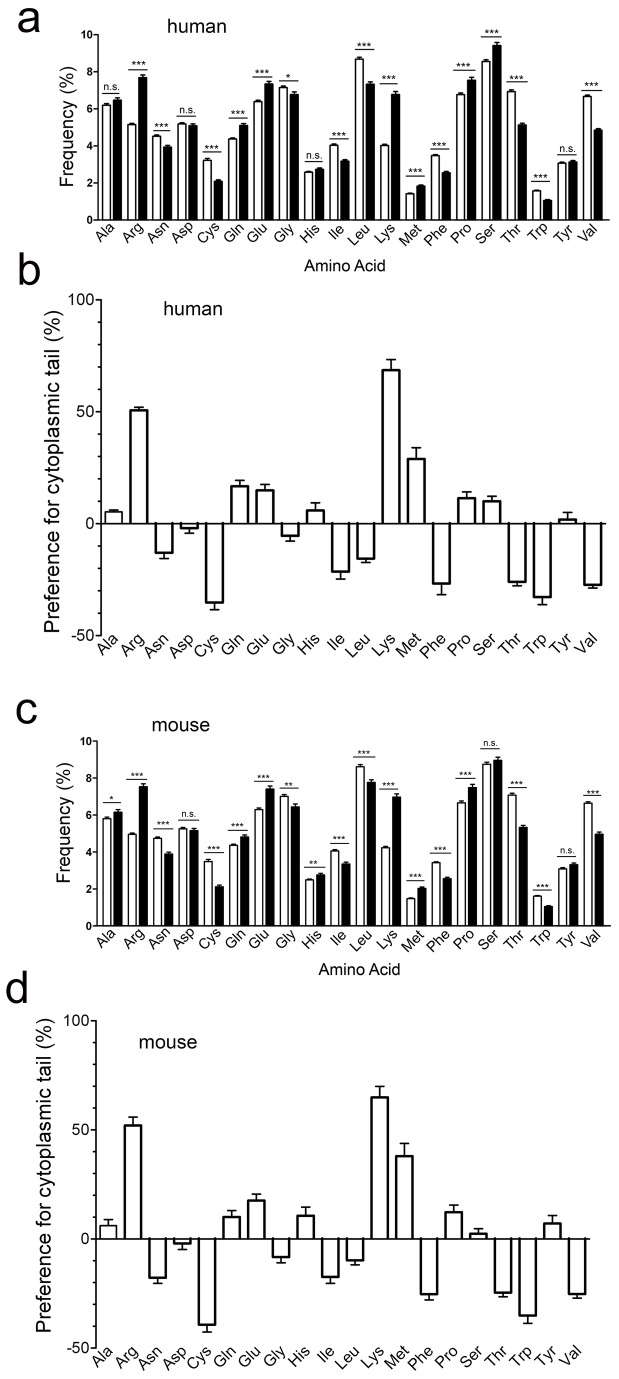
Amino acid frequency in type I transmembrane proteins. **a**, **c**) The frequency of individual amino acids in the extracellular (open bars) and cytoplasmic (solid bars) domains of 911 human (**a**) or 733 mouse (**c**) type I transmembrane proteins is shown. Significant differences in mean amino acid frequencies between extracellular and cytoplasmic domains are indicated: n.s., not significant; *, p ≤ 0.05; **, p ≤ 0.001; ***, p ≤ 0.0001. **b**, **d**) The relative preference of individual amino acids for the cytoplasmic domain relative to the extracellular domain of 911 human (**b**) or 733 mouse (**d**) type I transmembrane proteins was calculated as described in Materials and Methods. Positive values indicate a preference for the cytoplasmic domain.

To explore other determinants that may explain the ability of the B7 cytoplasmic tail to enhance the rate of intracellular transport, we examined the frequency of amino acids present in the extracellular or cytoplasmic domains of human type I transmembrane proteins. We found a significant bias for charged amino acids in the cytoplasmic domains of type I transmembrane proteins as compared to the extracellular domain of the same proteins (Figure 12a). Likewise, there was also a strong preference for arginine (7.69 ± 0.15% for cytoplasmic tails vs. 5.15 ± 0.07% for extracellular domains) and lysine (6.78 ± 0.15 for cytoplasmic tails vs. 4.02 ± 0.07 for extracellular domains) residues in mouse type I transmembrane proteins (Figure 12b). Methionine, glutamine, glutamic acid and proline were also overrepresented in both human (Figure 12c) and mouse (Figure 12d) cytoplasmic domains of type I transmembrane proteins.

**Figure 13:**
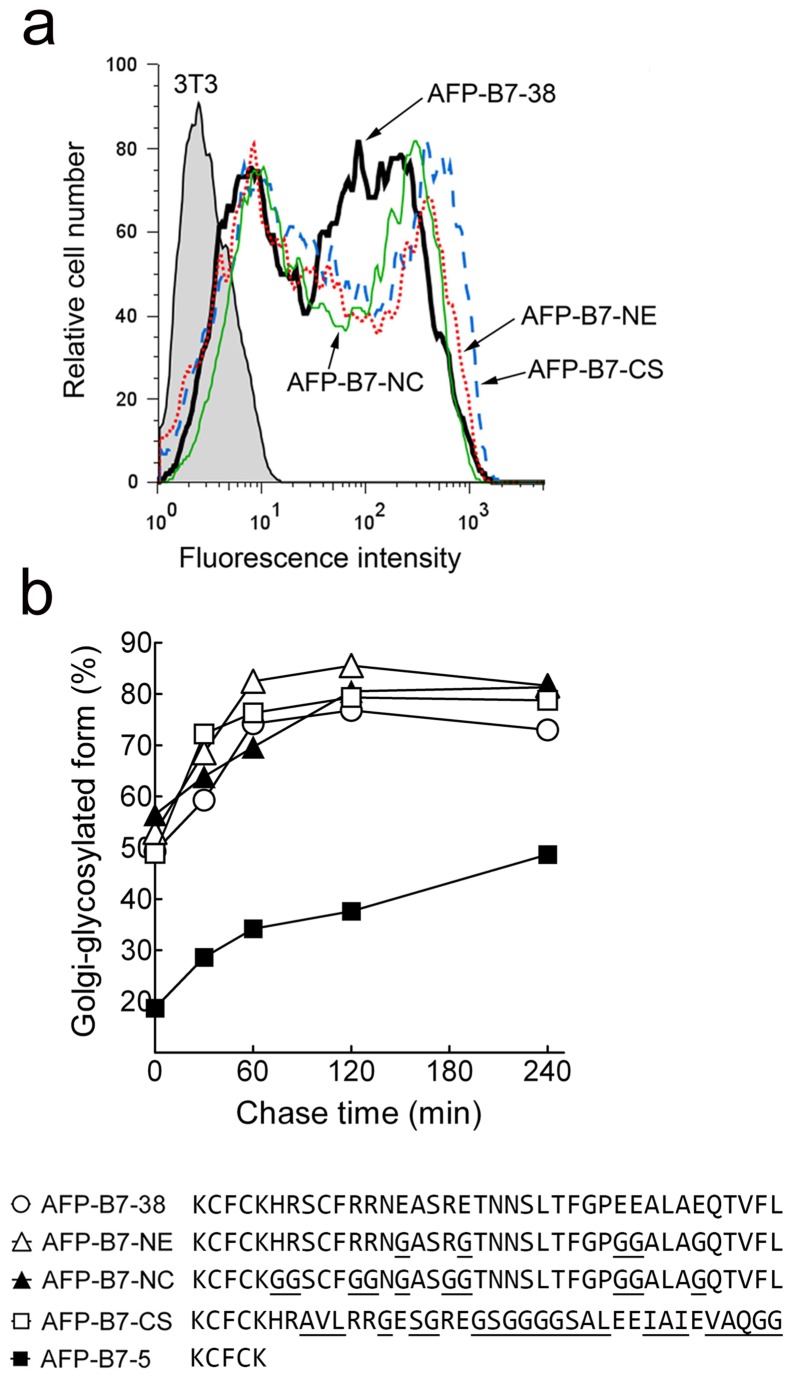
Charged amino acids in the B7 cytoplasmic tail are not responsible for effective intracellular transport. **a**) Acidic or both acidic and basic amino acids were replaced with glycine residues in AFP-B7-NE and AFP-B7-NC, respectively, whereas all amino acids except the charged amino acids were altered in AFP-B7-CS. The expression of chimeric proteins on the surface of transiently-transfected 3T3 cells was determined by flow cytometry. **b**) The percentage of intracellular Golgi-glycosylated AFP chimeras in relation to total intracellular AFP chimeric protein in ^35^S-methionine labeled cells at the indicated chase times is shown.

The high prevalence of charged amino acids in the cytoplasmic tail of type I transmembrane proteins prompted us to investigate whether charged amino acids contribute to intracellular transport of AFP-B7-38. We replaced acidic (AFP-B7-NE) or both acidic and basic amino acids (AFP-B7-NC) with glycine residues in the B7 cytoplasmic domain. AFP-B7-NE and AFP-B7-NC were highly expressed on 3T3 cells (Figure 13a) and were rapidly transported to the Golgi (Figure 13b). On the other hand, AFP-B7-CS, in which all non-charged amino acids were mutated, was also rapidly transported to the Golgi (Figure 13b). We conclude that neither specific charged nor non-charged amino acids in the B7 cytoplasmic domain are required for efficient intracellular transport.

### Predicted cytoplasmic secondary structure correlates with intracellular transport rate

**Figure 14:**
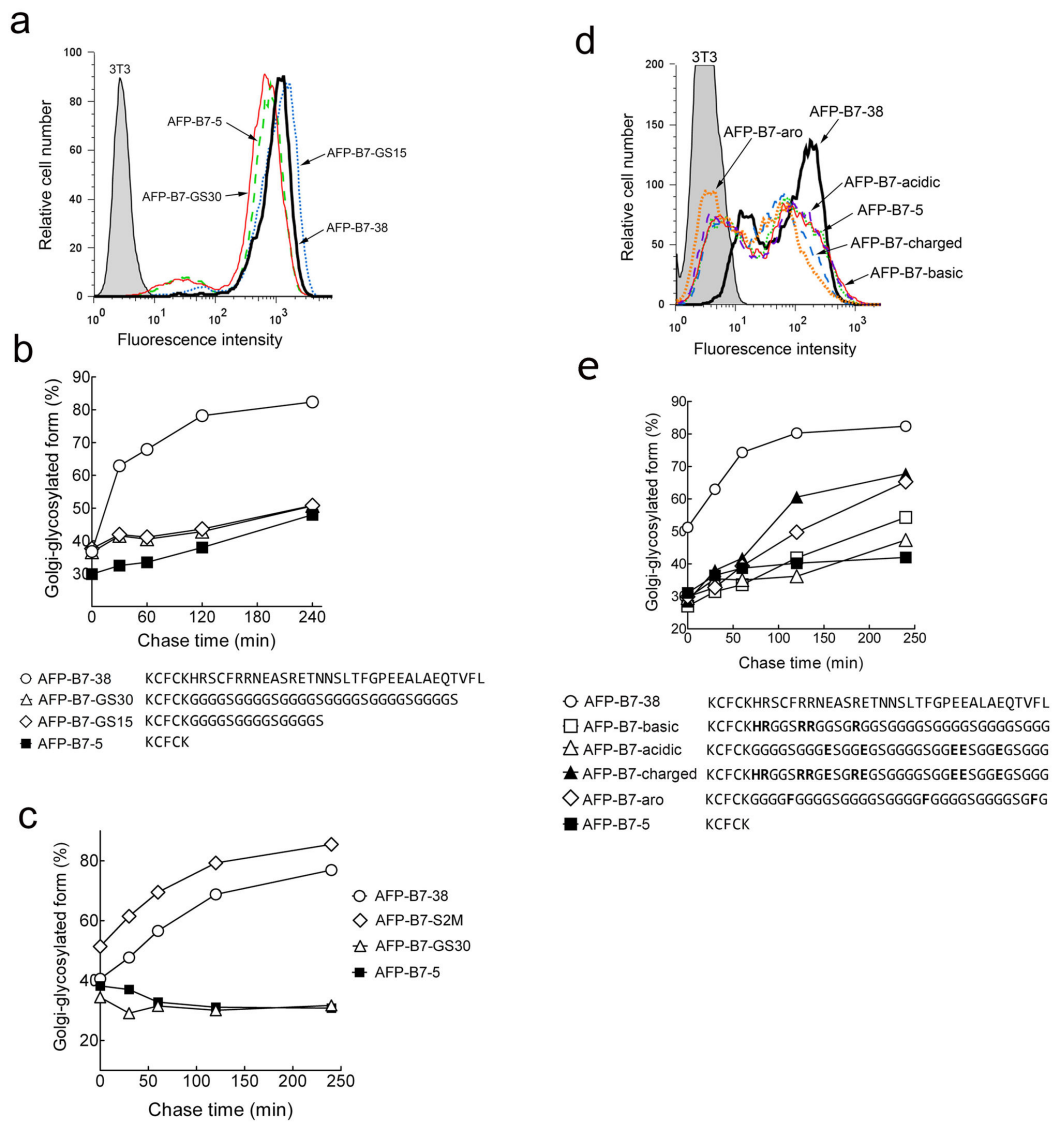
Intracellular transport rates of AFP chimeric proteins with flexible cytoplasmic domains. **a**) Non-structured cytoplasmic tails were generated by appending GGGGS repeats of 15 or 30 residues to AFP-B7-5 to generate AFP-B7-GS15 or AFP-B7-GS30, respectively. The expression of AFP-B7-38, AFP-B7-GS15, AFP-B7-GS30 and AFP-B7-5 on the surface of stably transfected 3T3 cells was determined by flow cytometry. **b**) The percentage of intracellular Golgi-glycosylated AFP chimeric protein relative to total intracellular AFP chimeric protein as a function of time is shown. **c**) The percentage of intracellular Golgi-glycosylated AFP chimeric protein relative to total intracellular AFP chimeric protein as a function of time is shown in stably transfected HEK293 cells. **d**) The basic (AFP-B7-basic), acidic (AFP-B7-acidic), charged (AFP-B7-charged) or aromatic (AFP-B7-aro) amino acids present in the B7 cytoplasmic domain were introduced into the same positions in a chimeric protein possessing GGGGS repeats in the cytoplasmic tail. The expression of chimeric proteins on the surface of transiently-transfected 3T3 cells was determined by flow cytometry. **e**) The percentage of intracellular Golgi-glycosylated AFP chimeric protein relative to total intracellular AFP chimeric protein as a function of time is shown.

To test a connection between secondary structure and transport rate, we generated chimeric proteins with highly flexible, unstructured polypeptides [90] of 30 (AFP-B7-GS30) or 15 (AFP-B7-GS15) amino acids appended to AFP-B7-5. Although both AFP-B7-GS30 and AFP-B7-GS15 were expressed on transfected 3T3 cells (Figure 14a), these proteins were slowly transported to the Golgi (Figure 14b). To verify that these results were not restricted to 3T3 fibroblasts, we stably expressed key AFP chimeric proteins in HEK293 cells and measured their rate of intracellular transport by pulse-chase analysis. In agreement with the results found for 3T3 fibroblasts, AFP-B7-38 and AFP-B7-S2M, which contained either the full length cytoplasmic tail or a scrambled B7 cytoplasmic tail, respectively, were rapidly transported to the Golgi whereas AFP-B7-5 and AFP-B7-GS30, which possess a truncated tail or a highly flexible unstructured cytoplasmic tail, were more slowly transported to the Golgi (Figure 14c).

**Figure 15:**
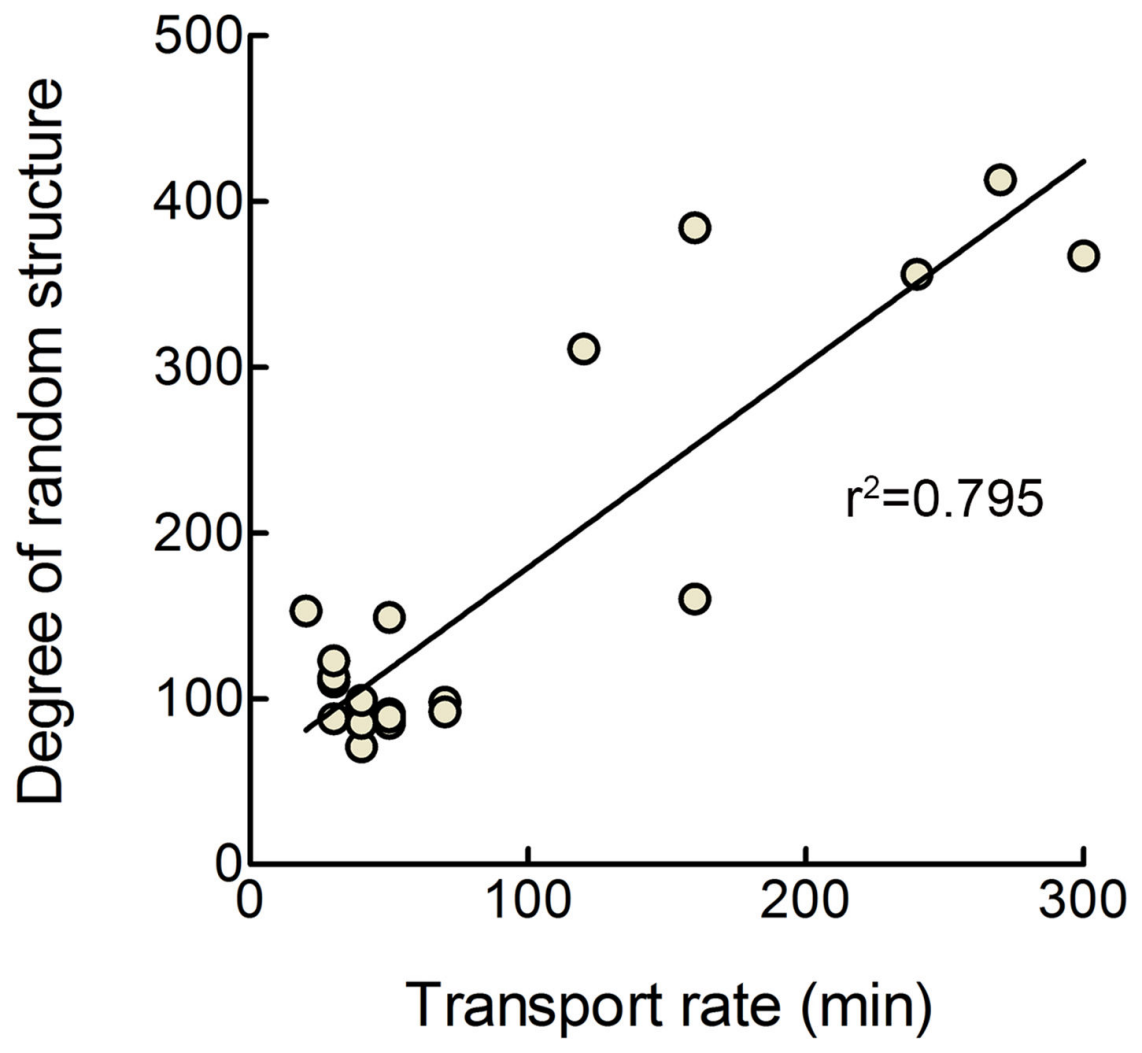
Correlation between predicted secondary structure of cytoplasmic tails and intracellular transport rate. Relative cytoplasmic tail structure (degree of random structure) was calculated by dividing the predicted percentage of random coil structure by the sum of the predicted percentage of helical and extended strand structures in the cytoplasmic tails of AFP chimeric proteins as calculated by the secondary structure prediction program SSPro 2.01. A large value of this metric corresponds to a predicted lack of ordered structure. The relative transport rates of AFP chimeric proteins (transport rate) were estimated by calculating the time required for 50% of the chimeric protein to attain the Golgi glycosylated form (complex carbohydrate) from the pulse-chase experiments. The best fit linear regression line for the data is also shown (r^2^ = 0.795).

Introduction of basic (AFP-B7-basic), acidic (AFP-B7-acidic), aromatic (AFP-B7-aro) or all of the corresponding charged amino acids (AFP-B7-charged) present in the B7 cytoplasmic domain into the corresponding positions in a flexible, unstructured cytoplasmic tail allowed expression on transiently-transfected 3T3 cells (Figure 14d) but did not greatly enhance the rate of intracellular transport (Figure 14e). Modeling of these chimeric proteins using four secondary structure prediction algorithms (SSPro 2.01, PSIPred, SOPMA and YASPIN) consistently predicted that these cytoplasmic tails largely display a random coil structure. We then modeled all cytoplasmic tails investigated in our study using these programs. To investigate a possible link between cytoplasmic tail structure and intracellular transport rate, we examined various metrics for cytoplasmic tail structure and intracellular transport rate. The best correlation was observed when we defined a metric for relative cytoplasmic tail order by dividing the predicted percentage of random coil by the sum of the predicted percentages of helical and extended strand structures in the cytoplasmic tails. A large value of this metric corresponds to a lack of secondary structure. A metric for the relative transport rates of the proteins was also estimated by calculating the time required for 50% of the chimeric protein to attain the Golgi glycosylated form (complex carbohydrate) from the results of pulse-chase experiments. A significant (p < 0.0001) correlation (r^2^ = 0.795) was observed between slower intracellular transport rate and the degree of random coli-like structure in the cytoplasmic tails using the secondary structures predictions from SSPro 2.01 (Figure 15). Significant (p < 0.0001) correlations between the presence of coli-like structure in cytoplasmic tails predicted using PSIPred, SOPMA and YASPIN and intracellular transport rates were also observed (r^2^ = 0.726, 0.722 and 0.781, respectively). Thus, we find that the rate of intracellular transport of chimeric proteins correlates with the predicted secondary structure of the cytoplasmic domain rather than with specific ER export motifs.

Cytoplasmic tail structure may also be involved in accelerated intracellular transport ascribed to some previously reported ER export motifs. For example, the six amino acid motif YTDIEM in the VSV G protein cytoplasmic tail has been shown to act as an ER export motif, yet replacement of this motif with 13 alanine residues, which were predicted to form an alpha-helical structure, resulted in efficient ER exit [68]. By contrast, replacement of the YTDIEM motif with glycine residues, which are predicted to form a disordered structure, resulted in slower ER exit, consistent with a link between secondary structure and anterograde transport. The FxxxxxxLL motif in the cytoplasmic domains of the α2B-adrenergic receptor and angiotensin II type 1A receptors are also predicted to from an amphipathic helix, which might be important for ER exit [73]. A conformational Golgi export signal formed by amino acids at the N- and C-terminal domains of Kir2.1 interacts with the AP1 adaptin complex for export to the cell surface [91]. Likewise, the COP II complex was found to recognize a conformational epitope on Sec22p rather than a specific linear amino acid sequence [25,92].

We did not determine the component that interacts with the B7 cytoplasmic tail. However, the slow intracellular transport of B7 chimeric proteins observed in cells expressing dominant negative forms of Sar1 and Rab1 is consistent with ER export via classical COPII-mediated transport. It seems likely that the B7 cytoplasmic tail may interact with Sec24, which is the principle adapter of the COP II complex responsible for binding to ER export motifs [93,94]. Consistent with this notion, a conformational epitope on Sec22p was previously demonstrated to be recognized by the C-site of Sec24p in the COP II complex [25,92]. It will be important in future studies to determine if one of the four isoforms of Sec24 can selectively bind to structural determinants in the cytoplasmic tail of B7 and other proteins [95].

## Conclusions

The B7 cytoplasmic domain can enhance the intracellular transport and surface expression of chimeric proteins. However, mutagenesis, deletion and shuffling studies demonstrated that the B7 cytoplasmic domain does not contain a linear ER export motif. Rather, faster intracellular transport correlated with the presence of ordered structure in cytoplasmic domains. Thus, appendage of a flexible, non-structured cytoplasmic domain resulted in about fourteen fold slower transport of chimeric proteins to the Golgi as compared to proteins with “structured” cytoplasmic tails. We speculate that proteins possessing structured cytoplasmic tails, typified by AFP-B7-38, AFP-B7-S1, AFP-B7-S2M, AFP-B7-NE, AFP-B7-NC and AFP-B7-CS, interact with the cellular transport machinery, presumable sec 24p in the COPII complex, for accelerated trafficking to the Golgi and cell surface. On the other hand, proteins with truncated cytoplasmic tails, typified by AFP-B7-5, or unstructured cytoplasmic tails, such as AFP-B7-GS-30, are likely unable to interact with the COPII machinery and therefore traffic to the plasma membrane via slower “bulk flow”.

Our examination of a large number of cytoplasmic tails revealed that many previously reported ER export motifs are rarely found in the cytoplasmic tail of type I transmembrane proteins or are found at similar frequencies as expected by chance. Conformational motifs may therefore be more commonly involved that previously thought in regulating the intracellular transport of transmembrane proteins. Increased understanding of the role of cytoplasmic domain structure on intracellular transport may help improve the design of chimeric surface proteins for diverse therapeutic applications [4-13] and promote new therapies for diseases associated with cytoplasmic domain mutations [96-101].

Our results suggest that the cytoplasmic tail of chimeric proteins designed for mammalian surface display should 1) possess a minimal length of about five amino acids for stable integration in membranes, 2) possess sufficient length (

> 30 amino acids) to accelerate active transport to the Golgi, and 3) avoid stretches of random-coil structure. These guidelines are most important for proteins with rapid turnover on the cell surface, such as observed for single-chain antibodies in our study, where intracellular transport appears to be rate-limiting for achieving high levels of chimeric proteins on the plasma membrane.

## Supporting Information

Table S1 **Primers used to construct chimeric proteins.**
(DOCX)

Table S2 **Amino acid sequence of chimeric protein cytoplasmic tails.**
(DOCX)

Table S3 **Amino acid and nucleotide sequences of juxtamembrane domains of GFP chimeric proteins used for glycosylation mapping.**
(DOCX)
