# Supplementary material for: The B7-1 Cytoplasmic Tail Enhances Intracellular Transport and Mammalian Cell Surface Display of Chimeric Proteins in the Absence of a Linear ER Export Motif
Source: PLoS One. 2013 Sep 20;8(9):e75084. doi: 10.1371/journal.pone.0075084 (PMC3779271; doi:10.1371/journal.pone.0075084)
Supplement: Table S1 — Primers used to construct chimeric proteins. (DOCX) [file pone.0075084.s001.docx]

**Table S1. Primers used to construct chimeric proteins**

| **Plasmid** | **Template** | **Primers** | **Primer sequences (5’ – 3’)** |
| --- | --- | --- | --- |
| p2C11-PDGFR-xstp | p2C11-PDGFR | T7 | TAATACGACTCACTATAGGG |
| p2C11-PDGFR-xstp | p2C11-PDGFR | R1 | GCGGCCTCGAGACGTGGCTTCTTCTGCCAAAG |
| p2C11-PDGFR-B7 | 2C11-PDGFR-B7 | F2 | CGGCGGTCGACAAATGCTTCTGTAAGCACAG |
| p2C11-PDGFR-B7 | 2C11-PDGFR-B7 | R2 | CTCATTTTATTAGGAAAGGA |
| pAFP-B7-1 | p2C11-B7-38 | T7 | TAATACGACTCACTATAGGG |
| pAFP-B7-1 | p2C11-B7-38 | R3 | TGCTTCTCGAGCTATTTGATGATGACAACGATGAC |
| pAFP-B7-5 | p2C11-B7-38 | T7 | TAATACGACTCACTATAGGG |
| pAFP-B7-5 | p2C11-B7-38 | R4 | AAACACTCGAGCTACTTACAGAAGCATTTGATGAT |
| pAFP-B7-10 | p2C11-B7-38 | T7 | TAATACGACTCACTATAGGG |
| pAFP-B7-10 | p2C11-B7-38 | R5 | GCCTCCTCGAGCTAGAAACAGCTTCTGTGCTTACA |
| pAFP-B7-19 | p2C11-B7-38 | T7 | TAATACGACTCACTATAGGG |
| pAFP-B7-19 | p2C11-B7-38 | R6 | GTAAGCTCGAGCTATGTTTCTCTGCTTGCCTCATT |
| pAFP-B7-28 | p2C11-B7-38 | T7 | TAATACGACTCACTATAGGG |
| pAFP-B7-28 | p2C11-B7-38 | R7 | TCAGCCTCGAGCTATTCAGGCCCGAAGGTAAGGCT |
| pAGP-B7-GS15 | p2C11-B7-5 | T7 | TAATACGACTCACTATAGGG |
| pAGP-B7-GS15 | p2C11-B7-5 | G4S-1 | ATTCTCGAGTTAAGATCCACCTCCTCCACTTCCACCTCCACCTGATCCACCACCTCCCTTACAGAAGCATTTGATGATGAC |
| pAFP-B7-GS30 | pAGP-B7-GS15 | T7 | TAATACGACTCACTATAGGG |
| pAFP-B7-GS30 | pAGP-B7-GS15 | G4S-2 | ATTCTCGAGTTAACTTCCACCACCACCGCTTCCACCACCTCCTGAACCTCCTCCACCAGATCCACCTCCTCCACTTCC |
| pAFP-B7-M1 | p2C11-B7-38 | M1-F | GCTTCTGTAAGCACAGAAGCGGTGTCGGAGGAAGTGAGGCAAGCAGAGAAAC |
| pAFP-B7-M1 | p2C11-B7-38 | M1-R | GTTTCTCTGCTTGCCTCACTTCCTCCGCCACCGCTTCTGTGCTTACAGAAGC |
| pAFP-B7-M2 | p2C11-B7-38 | M2-F | CAGCCTTACCTTCGGGCCTGGAGGAGGAGTATCTGAACAGACCGTCTTCC |
| pAFP-B7-M2 | p2C11-B7-38 | M2-R | GGAAGACGGTCTGTTCAGATCCTCCTCCTCCAGGCCCGAAGGTAAGGCTG |
| pAFP-B7-M3 | p2C11-B7-38 | M3-F | GAAGCAAGCAGAGAAGCAAACGCCGCCGCTACCTTCGGGCT GAA |
| pAFP-B7-M3 | p2C11-B7-38 | M3-R | TTCAGGCCCGAAGGTAGCGGCGGCGTTTGCTTCTCTGCTTGCTTC |
| pAFP-B7-M4 | p2C11-B7-38 | M4-F | GAGGCAAGCAGAGAAACAGCCGCCGCCGCTACCTTCGGGCCTGAAGAAG |
| pAFP-B7-M4 | p2C11-B7-38 | M4-R | CTTCAGGCCCGAAGGTAGCGGCGGCGGCTGTTTCTCTGCTTGCCTC |
| pAFP-B7-M5 | p2C11-B7-38 | M5-F | GAAACAAACAACAGCCTTGCCGCCGCGCCTGAAGAAGCATTAGCT |
| pAFP-B7-M5 | p2C11-B7-38 | M5-R | AGCTAATGCTTCTTCAGGCGCGGCGGCAAGGCTGTTGTTTGTTCC |
| pAFP-B7-M6 | p2C11-B7-38 | M6-F | AACAGCCTTACCTTCGGGGCTGCAGCAGCATTAGCTGAACAGACC |
| pAFP-B7-M6 | p2C11-B7-38 | M6-R | GGTCTGTTCAGCTAATGCTGCTGCAGCCCCGAAGGTAAGGCTGTT |
| pAFP-B7-M7 | p2C11-B7-38 | M7-F | ACCTTCGGGCCTGAAGAAGCAGCAGCTGAACAGACCGTCTTCCTT |
| pAFP-B7-M7 | p2C11-B7-38 | M7-R | AAGGAAGACGGTCTGTTCAGCTGCTGCTTCTTCAGGCCCGAAGGT |
| pAFP-B7-M8 | p2C11-B7-38 | M8-F | CCTGAAGAAGCATTAGCTGCAGCGGCCGTCTTCCTTTAGCTCGAA |
| pAFP-B7-M8 | p2C11-B7-38 | M8-R | TTCGAGCTAAAGGAAGACGGCCGCTGCAGCTAATGCTTCTTCAGG |
| pAFP-B7-M9 | p2C11-B7-38 | M9-F | GCATTAGCTGAACAGACCGCCGCCGCTTAGCTCGAAACATCGATA |
| pAFP-B7-M9 | p2C11-B7-38 | M9-R | TATCGATGTTTCGAGCTAAGCGGCGGCGGTCTGTTCAGCTAATGC |
| pAFP-B7-A5 | p2C11-B7-38 | T7 | TAATACGACTCACTATAGGG |
| pAFP-B7-A5 | p2C11-B7-38 | M10-R | ATATCGATCTAAGCAGCAGCAGCAGCGATGATGACAACGAT |
| pAFP-B7(Δ6-20) | p2C11-B7-38 | D15-F | GGT AAG GCT GTT GTT CTT ACA GAA GCA TTT |
| pAFP-B7(Δ6-20) | p2C11-B7-38 | D15-R | AAA TGC TTC TGT AAG AAC AAC AGC CTT ACC |
| pAFP-B7-S1 | pAFP-B7-5 | S1-1 | ATTGAATCTCTTACAGAAGCATTTGATGATGACAACGATGACGACGAC |
| pAFP-B7-S1 | pAFP-B7-5 | S1-2 | GCTTCTGTAAGAGATTCAATAGAGCAGAGAGCTGTACACACGAAAATA |
| pAFP-B7-S1 | pAFP-B7-5 | S1-3 | GTTCTCTGTTCAAGGCTTTCTCTCCCGCTATTTTCGTGTGTACAGCTC |
| pAFP-B7-S1 | pAFP-B7-5 | S1-4 | GAAAGCCTTGAACAGAGAACATTCAATCTTTTCGTCTTACCTGCAGAA |
| pAFP-B7-S1 | pAFP-B7-5 | S1-5 | TCTCTATCGATGTTTCGAGCTATGCGGTTTCTGCAGGTAAGACGAAAA |
| pAFP-B7-S2 | pAFP-B7-5 | S2-1 | TTCTCTGCTCTTACAGAAGCATTTGATGATGACAACGATGACGACGAC |
| pAFP-B7-S2 | pAFP-B7-5 | S2-2 | GCTTCTGTAAGAGCAGAGAAAGAAATCTTGGGGAAGCTACCCTTAGAT |
| pAFP-B7-S2 | pAFP-B7-5 | S2-3 | AACTGAAGTTCGAAGCTTGTGCTATTGAATCTAAGGGTAGCTTCCCCA |
| pAFP-B7-S2 | pAFP-B7-5 | S2-4 | ACAAGCTTCGAACTTCAGTTCCACTGCAGAGCAGAGAACACCCCTGCT |
| pAFP-B7-S2 | pAFP-B7-5 | S2-5 | TCTCTATCGATGTTTCGAGCTAGACCTCAGCAGGGGTGTTCTCTGCTCT |
| pAFP-B7-NE | pAFP-B7-5 | NE-1 | GCTTCTGTGCTTACAGAAGCATTTGATGATGACAACGATGACGACGAC |
| pAFP-B7-NE | pAFP-B7-5 | NE-2 | GCTTCTGTAAGCACAGAAGCTGTTTCAGAAGAAATGGCGCAAGCAGAG |
| pAFP-B7-NE | pAFP-B7-5 | NE-3 | GGCCCGAAGGTAAGGCTGTTGTTTGTACCTCTGCTTGCGCCATTTCTT |
| pAFP-B7-NE | pAFP-B7-5 | NE-4 | AACAGCCTTACCTTCGGGCCTGGAGGCGCATTAGTCGGTCAGACCGTC |
| pAFP-B7-NE | pAFP-B7-5 | NE-5 | TCTCTATCGATGTTTCGAGCTAAAGGAAGACGGTCTGACCGACTAATG |
| pAFP-B7-NC | pAFP-B7-5 | NC-1 | GCTCCCTCCCTTACAGAAGCATTTGATGATGACAACGATGACGACGAC |
| pAFP-B7-NC | pAFP-B7-5 | NC-2 | GCTTCTGTAAGGGAGGGAGCTGTTTCGGTGGCAATGGCGCAAGCGGAG |
| pAFP-B7-NC | pAFP-B7-5 | NC-3 | GGCCCGAAGGTAAGGCTGTTGTTTGTACCTCCGCTTGCGCCATTGCCA |
| pAFP-B7-NC | pAFP-B7-5 | NC-4 | AACAGCCTTACCTTCGGGCCTGGAGGCGCATTAGTCGGTCAGACCGTC |
| pAFP-B7-NC | pAFP-B7-5 | NC-5 | TCTCTATCGATGTTTCGAGCTAAAGGAAGACGGTCTGACCGACTAATG |
| pAFP-B7-CS | pAFP-B7-5 | CS-1 | TGCTCTGTGCTTACAGAAGCATTTGATGATGACAACGATGACGACGAC |
| pAFP-B7-CS | pAFP-B7-5 | CS-2 | GCTTCTGTAAGCACAGAGCAGTCCTTAGAAGAGGCGAAAGTGGTAGAG |
| pAFP-B7-CS | pAFP-B7-5 | CS-3 | AGTGCAGAGCCACCACCTCCACTCCCTTCTCTACCACTTTCGCCTCTT |
| pAFP-B7-CS | pAFP-B7-5 | CS-4 | GGAGGTGGTGGCTCTGCACTTGAAGAGATCGCTATAGAAGTCGCTCAG |
| pAFP-B7-CS | pAFP-B7-5 | CS-5 | TCTCTATCGATGTTTCGAGCTAGCCACCCTGAGCGACTTCTATAGCGA |
