# Supplementary material for: The B7-1 Cytoplasmic Tail Enhances Intracellular Transport and Mammalian Cell Surface Display of Chimeric Proteins in the Absence of a Linear ER Export Motif
Source: PLoS One. 2013 Sep 20;8(9):e75084. doi: 10.1371/journal.pone.0075084 (PMC3779271; doi:10.1371/journal.pone.0075084)
Supplement: Table S2 — Amino acid sequence of chimeric protein cytoplasmic tails. (DOCX) [file pone.0075084.s002.docx]

**Table S2. Amino acid sequence of chimeric protein cytoplasmic tails**

| **Chimeric protein** | **CT amino acid sequence** |
| --- | --- |
| AFP-B7-38 | KCFCKHRSCFRRNEASRETNNSLTFGPEEALAEQTVFL |
| AFP-B7-28 | KCFCKHRSCFRRNEASRETNNSLTFGPE |
| AFP-B7-19 | KCFCKHRSCFRRNEASRET |
| AFP-B7-10 | KCFCKHRSCF |
| AFP-B7-5 | KCFCK |
| AFP-B7-1 | K |
| AFP-B7-AAAAA | AAAAA |
| AFP-PDGFR | WQKKPR |
| AFP-PDGFR-B7 | WQKKPRLDKCFCKHRSCFRRNEASRETNNSLTFGPEEALAEQTVFL |
| AFP-B7-M1 | KCFCKHRSGVGGSEASRETNNSLTFGPEEALAEQTVFL |
| AFP-B7-M2 | KCFCKHRSCFRRNEASRETNNSLTFGPGGGVSEQTVFL |
| AFP-B7-M3 | KCFCKHRSCFRRNEASREANAAATFGPEEALAEQTVFL |
| AFP-B7-M4 | KCFCKHRSCFRRNEASRETAAALTFGPEEALAEQTVFL |
| AFP-B7-M5 | KCFCKHRSCFRRNEASRETNNSAAAAPEEALAEQTVFL |
| AFP-B7-M6 | KCFCKHRSCFRRNEASRETNNSLTFGAAAALAEQTVFL |
| AFP-B7-M7 | KCFCKHRSCFRRNEASRETNNSLTFGPEEAAAEQTVFL |
| AFP-B7-M8 | KCFCKHRSCFRRNEASRETNNSLTFGPEEALAAAAVFL |
| AFP-B7-M9 | KCFCKHRSCFRRNEASRETNNSLTFGPEEALAEQTAAA |
| AFP-B7(Δ6-20) | KCFCKNNSLTFGPEEALAEQTVFL |
| AGP-B7-GS15 | KCFCKGGGGSGGGGSGGGGS |
| AFP-B7-GS30 | KCFCKGGGGSGGGGSGGGSGGGGSGGGGSGGGGS |
| AFP-B7-S1 | KCFCKRFNRAESCTHENSGRESLEQRTFNLFVLPAETA |
| AFP-B7-S2 | KCFCKSRERNLGEATLRFNSTSFELQFHCRAENTPAEV |
| AFP-B7-S2M | KCFCKSGEGNLGEATLRFNSTSFELQFHCRAENTPAEV |
| AFP-B7-NE | KCFCKHRSCFRRNGASRGTNNSLTFGPGGALAGQTVFL |
| AFP-B7-NC | KCFCKGGSCFGGNGASGGTNNSLTFGPGGALAGQTVFL |
| AFP-B7-CS | KCFCKHRAVLRRGESGREGSGGGGSALEEIAIEVAQGG |
| AFP-B7-basic | KCFCK HRGGSRRGGSGRGGSGGGGSGGGGSGGGGSGGG |
| AFP-B7-acidic | KCFCK GGGGSGGGESGGEGSGGGGSGGEESGGEGSGGG |
| AFP-B7-aro | KCFCK GGGGFGGGGSGGGGSGGGGFGGGGSGGGGSGFG |
| AFP-B7-charged | KCFCK HRGGSRRGESGREGSGGGGSGGEESGGEGSGGG |
