# Supplementary material for: The B7-1 Cytoplasmic Tail Enhances Intracellular Transport and Mammalian Cell Surface Display of Chimeric Proteins in the Absence of a Linear ER Export Motif
Source: PLoS One. 2013 Sep 20;8(9):e75084. doi: 10.1371/journal.pone.0075084 (PMC3779271; doi:10.1371/journal.pone.0075084)
Supplement: Table S3 — Amino acid and nucleotide sequences of juxtamembrane domains of GFP chimeric proteins used for glycosylation mapping. (DOCX) [file pone.0075084.s003.docx]

**Table S3. Amino acid and nucleotide sequences of juxtamembrane domains of GFP chimeric proteins used for glycosylation mapping.**

| **Chimeric**  **protein** | **Linker length**  **(amino acids)** | **Linker amino acid and nucleotide sequences** |
| --- | --- | --- |
| GFP-0-B7-38 | NA | Val Asp Pro Pro Glu Asp Pro Pro Asp Ser Lys Asn **Thr Leu Val** |
| GFP-0-B7-38 | NA | gtc gac ccc cca gaa gac cct cct gat agc aag aac aca cct gtg |
| GFP-8-B7-38 | 8 | Val Asp Pro Pro Glu **Asn** Arg Thr Ser Asp Ser Lys Asn **Thr Leu Val** |
| GFP-8-B7-38 | 8 | gtc gac ccc cca gaa aac cgt acg agc gat agc aag aac aca cct gtg |
| GFP-10-B7-38 | 10 | Val Asp Pro Pro Glu **Asn** Arg Thr Ser Glu Gln Asp Ser Lys Asn **Thr Leu Val** |
| GFP-10-B7-38 | 10 | gtc gac ccc cca gaa aac cgt acg agc gaa caa gat agc aag aac aca cct gtc |
| GFP-12-B7-38 | 12 | Val Asp Pro Pro Glu **Asn** Arg Thr Ser Glu Asn Lys Gln Asp Ser Lys Asn **Thr Leu Val** |
| GFP-12-B7-38 | 12 | gtc gac ccc cca gaa aac cgt acg agc gaa aac aag caa gat agc aag aac aca cct gtc |
| GFP-14-B7-38 | 14 | Val Asp Pro Pro Glu **Asn** Arg Thr Ser Glu Asn Ser Asp Lys Gln Asp Ser Lys Asn **Thr Leu Val** |
| GFP-14-B7-38 | 14 | gtc gac ccc cca gaa aac cgt acg agc gaa aac agc gat aag caa gat agc aag aac aca cct gtc |
| GFP-16-B7-38 | 16 | Val Asp Pro Pro Glu **Asn** Arg Thr Ser Glu Asn Ser Glu Gln Asp Lys Gln Asp Ser Lys Asn **Thr Leu Val** |
| GFP-16-B7-38 | 16 | gtc gac ccc cca gaa aac cgt acg agc gaa aac agc gaa caa gat aag caa gat agc aag aac aca cct gtc |

The original chimeric protein possessed 10 amino acids between the Sal I site at the C-terminus of GFP and the start of the TM domain (in blue). Oligonucleotide primers coding the indicated spacer domains with a single N-linked glycosylation site (red) were introduced between the Sal I (gtcgac) and Ale I (cacnnnngtc) restriction enzyme sites (underlined).
